# Functional Evaluations of Genes Disrupted in Patients with Tourette’s Disorder

**DOI:** 10.3389/fpsyt.2016.00011

**Published:** 2016-02-09

**Authors:** Nawei Sun, Jay A. Tischfield, Robert A. King, Gary A. Heiman

**Affiliations:** ^1^Department of Genetics, Rutgers University, Piscataway, NJ, USA; ^2^Human Genetics Institute of New Jersey, Piscataway, NJ, USA; ^3^Child Study Center, Yale School of Medicine, New Haven, CT, USA

**Keywords:** Tourette’s disorder, functional studies, genes, neurobiology, induced pluripotent stem cells

## Abstract

Tourette’s disorder (TD) is a highly heritable neurodevelopmental disorder with complex genetic architecture and unclear neuropathology. Disruptions of particular genes have been identified in subsets of TD patients. However, none of the findings have been replicated, probably due to the complex and heterogeneous genetic architecture of TD that involves both common and rare variants. To understand the etiology of TD, functional analyses are required to characterize the molecular and cellular consequences caused by mutations in candidate genes. Such molecular and cellular alterations may converge into common biological pathways underlying the heterogeneous genetic etiology of TD patients. Herein, we review specific genes implicated in TD etiology, discuss the functions of these genes in the mammalian central nervous system and the corresponding behavioral anomalies exhibited in animal models, and importantly, review functional analyses that can be performed to evaluate the role(s) that the genetic disruptions might play in TD. Specifically, the functional assays include novel cell culture systems, genome editing techniques, bioinformatics approaches, transcriptomic analyses, and genetically modified animal models applied or developed to study genes associated with TD or with other neurodevelopmental and neuropsychiatric disorders. By describing methods used to study diseases with genetic architecture similar to TD, we hope to develop a systematic framework for investigating the etiology of TD and related disorders.

## Introduction

Tourette’s Disorder (TD) is a childhood-onset neurodevelopment disorder characterized by the presence of both motor and vocal tics. Prevalence ranges from 1–3% and is found across many ethnic groups around the world (1). However, a recent meta-analysis of previous TD prevalence studies re-estimates the population prevalence of TD to be 0.3–0.9% (2). Males are affected three to four times more often than females (3–5). A high percentage of TD patients have comorbid conditions, most commonly attention-deficit/hyperactivity disorder (ADHD) and obsessive–compulsive disorder (OCD) and to a lesser extent autism spectrum disorders (ASDs).

Consistent evidence from family and twin studies suggest a significant genetic contribution to TD, most likely the result of complex and heterogeneous inheritance involving both common and rare variants, though most of specific findings still require replication. The neurobiological basis of TD is not well understood, but appears to involve alterations in the development, structure, and/or functioning of cortico-striato-thalamo-cortical (CSTC) circuits (6). Specific genes have been found to be associated with TD. It is unclear if mutations in these genes cause TD and, if so, how these alterations affect the function or structural development of the nervous system. Our focus is to review the neurobiology of TD, describe the biological functions of those genes previously associated with TD, and discuss the various functional analyses that are required for evaluating and establishing the pathogenicity of these putative genetic causal variants for TD.

## Neurobiology of Tourette’s Disorder

Alterations of the CSTC circuits are considered as the neuropathological basis of tic generation (6, 7). These alterations are apparent in functional and structural imaging studies (8), histopathological studies of specific neuronal populations (9), and defective inhibition in various electroneurophysiological experimental paradigms (10, 11). In addition to the male predominance, the developmental features of TD pose an explanatory challenge, with tics usually not appearing till 4–6 years of age and most often, but not always, improving spontaneously by late adolescence.

Neurotransmitter pathways that modulate the activity and the output of the CSTC circuits in the basal ganglia have been the focus of intensive investigation, driven in part by the quest for more effective pharmacological interventions. The most supported neurotransmitter dysregulation hypothesis in TD involves the hyperactivity or imbalance of the dopamine signaling in the striatum (12). Within the basal ganglia, dopamine is released to the striatum by dopaminergic neurons originating from the substantia nigra. In the striatum, the effect of dopamine on subsequent neural signal transmission is modulated by the striatal medium spiny neurons expressing either D1 or D2-like dopamine receptors (13). The dopamine hypothesis is supported by the clinical observation that dopamine D2 receptor antagonists effectively reduced tics in some TD patients (14, 15). Also, dopamine pathway dysregulation was reported in post-mortem TD brain samples (16, 17) and in living TD patients’ brain (12, 18).

Due to their excitatory and inhibitory effects within CSTC circuits, glutamatergic and GABAergic pathways have also been studied in TD. In post-mortem samples, lower levels of glutamate in subcortical brain regions were reported (19). However, it is unclear whether TD is associated with hyper- or hypo-glutamate levels (20). For the GABAergic pathway, an altered number and distribution of striatal GABAergic neurons were described in TD post-mortem brain samples (9, 21).

Disrupted serotonin signaling has been implicated in OCD, a common comorbid condition among TD patients. Selective serotonin reuptake inhibitors (SSRIs) have proven effective in reducing OCD symptoms (22) and are also used to treat TD patients with comorbid OCD (23). Interestingly, sequence variants at the serotonin transporter (SERT) gene were found in both OCD and TD patients (24), suggesting alterations in the serotonin pathway as one possible mechanism in the etiology of TD.

Until the recent discovery of a dominant negative non-sense mutation in the *HDC* gene co-segregating with TD in a large family, histaminergic (HA) neurotransmission was not considered a top candidate for TD etiology (25). However, other findings provide additional support for the involvement of HA neurotransmission in TD. For example, single nucleotide polymorphisms (SNPs) within the *HDC* gene region showed association with TD (26). Also, rare copy number variants (CNVs) found in TD patients were enriched in chromosomal regions harboring HA pathway genes (27). Furthermore, mice lacking the *Hdc* gene exhibited tic-like behaviors (28). Even though no evidence showed direct actions of serotonin and histamine on movements, it is proposed that serotonin and histamine pathways might indirectly regulate movements by modulating the dopamine system in the substantia nigra. In particular, both serotoninergic and HA innervations are observed in the substantia nigra (29, 30). Also, serotonin and histamine receptors are expressed on nigrostriatal dopaminergic neurons (30, 31).

Aside from the neurotransmitter dysregulation hypothesis in TD, developmental and neuroimmunological findings also provide a context for assessing the relevance of potential gene findings. Altered distribution of parvalbumin interneurons (21) and reduced numbers of parvalbumin and cholinergic interneurons in basal ganglia were observed in the post-mortem brain samples of TD patients (9), suggesting another possible, perhaps developmental, mechanism for alterations of CSTS circuits. Additionally, a dysregulated brain-immune system involving microglia cells was suggested to contribute to TD (32). Gene expressions of inflammatory factors were examined using post-mortem basal ganglia samples from TD patients and controls (33). An elevated expression of the *CD45* gene was observed in TD patients even though the elevation was not statistically significant. CD45 is a surface marker of microglia and its expression is increased due to the activation of microglia. In another study, transcriptome analysis of post-mortem striatum of TD patients and controls revealed upregulation of microglia-related genes (34).

## Genes Disrupted in Patients with Tourette’s Disorder

In this section, we will review 15 genes that have been associated in TD (Table 1); to suggest how we might move beyond association to establish a role in TD pathogenesis, we will examine what is known about the biological effects of these genes. We group these genes into several categories: (1) neurite outgrowth: *SLITRK1*; (2) histamine pathway: *HDC*; (3) serotonin pathway: *SERT, HTR1A HTR2B*; (4) glutamate pathway: *SLC1A3*; (5) synaptic signal transduction and cell-adhesion pathway: *NLGN4, CDH2, CNTNAP2/CASPR2, DPP6*; (6) mitochondrial functions: *IMMP2L, MRPL3*; and (7) genes associated with other diseases: *DNAJC13* [Parkinson’s disease (PD)], *OFCC1* (orofacial clefts), and *HCRTR2 or OX2R* (excessive daytime sleepiness). The diverse functions of these genes – ranging from neurotransmitter synthesis, neuronal migration, synaptic plasticity, cell adhesion, and protein transportation and synthesis – highlight the complexity of unraveling the pathogenesis of TD. However, in addition to the genetic disruptions discussed here, large structural variations, for example copy number variations (CNVs), have also been investigated in TD patients. Genes disrupted by these structural variants have been discovered and indicated as potential TD associated genes (35–37).

**Table 1:** **Genes disrupted in TD**.

Biological pathways	Gene name	Disruption(s) found in TD patients and references
Neurite outgrowth	*SLITRK1*	Inversion, frameshift variant, Var321 (38)
		Synonymous variant: 708C > T (39)
		3’-UTR variant: 3383G > A (43)
		3225 T > C (41)
		D397G (44)
Histamine	*HDC*	W317X (25)
		Intronic transition and synonymous variants: 426C > A, 1743G > A (55)
Serotonin	*SERT*	I425V and 5-HTTLPR (24)
	*HTR1A*	R219L (78)
	*HTR2B*	M63R, R449Q (79)
Glutamate	*SLC1A3/EAAT1*	E219D (88)
Synaptic signal transduction and cell adhesion	*NLGN4/NLGN4X*	Deletion across exon 4, 5 and 6 (99)
	*CDH2*	N706S, N845S (106)
	*CNTNAP2*	Intronic insertion (121)
	*DPP6*	Microdeletion at exon 1 (128)
Mitochondrial functions	*IMMP2L*	Translocation (141)
		*De novo* duplication (142)
		Translocation and cryptic deletion eliminated the exon 1–3 (143)
		Intragenic deletions (144)
	*MRPL3*	S75N (150)
Genes associated with other diseases	*DNAJC13* (Parkinson’s disease)	A2057S (150)
	*OFCC1* (orofacial clefts)	R129G and a novel variant at 5′-UTR (150)
	*HCRTR2/OX2R* (excessive daytime sleepiness)	P10S (162)

### Neurite Outgrowth

#### SLIT and NTRK-Like Family, Member 1 (*SLITRK1*)

In a TD patient with comorbid ADHD, a *de novo* chromosome 13 inversion was identified (38). The *SLITRK1* gene was mapped close to the breakpoints. Targeted sequencing of the *SLITRK1* gene identified a non-coding variant (var321) and a frameshift mutation (38) in another 174 unrelated TD patients but not in a large control sample. The frameshift mutation led to impaired dendrite growth in neurons and the var321 variant may cause reduced SLITRK1 protein expression (38). While the var321 and additional novel variants within *SLITRK1* were found in other TD patients, these associations were not replicated in subsequent studies (39–49).

Members of the SLITRK protein family are transmembrane proteins. They are structurally homologous to the SLIT and the TRK proteins, which are involved in axon guidance pathway and neurodevelopment (50). The *SLITRK1* gene is highly expressed in developing and mature neuronal tissues and promotes neurite outgrowth in culture (51). The SLITRK1 protein is localized to the post-synaptic densities and has been hypothesized to affect synapse formation at excitatory synapses through interactions with the pre-synaptic cellular adhesion molecule LAR-RPTP (52, 53). In *Slitrk1* knockout mice, although stereotypic behaviors were not observed, the mice exhibited anxiety-like and depression-like behaviors, which were attenuated by chemicals modulating noradrenergic neurotransmission (54). Neurochemical abnormalities were also detected in *Slitrk1* knockout mice: norepinephrine and its metabolites were significantly increased in the prefrontal cortex and the nucleus accumbens while choline and acetylcholine levels were significantly lower in the striatum (54). Taken together, the evidence suggests that the *SLITRK1* gene might play a role in neurochemical modulation.

### Histamine Pathway

#### Histidine Decarboxylase

Histamine neurotransmission was first linked to the etiology of TD when a rare non-sense mutation within the *HDC* gene was discovered in a multiplex family in which the father and all eight children were diagnosed with TD. The mutation resulted from a G to A transition at the ninth exon of the *HDC* gene and led to a premature stop codon (W317X) (25). The heterozygous W317X mutation co-segregated with all affected individuals in this family. The W317X mutation was not found in 3360 unrelated individuals unaffected with TD or another 720 TD patients, suggesting this is a very rare cause for TD. *In vitro* enzymatic assay demonstrated that the truncated protein produced by the mutation lost histidine decarboxylase (HDC) activity and had a dominant negative effect on the activity of wild-type HDC protein. After the initial finding, more TD patients were screened for mutations in the *HDC* gene in different studies. Only an intronic variant and two synonymous variants were identified in a study involving 120 TD patients (55). However, an association of the *HDC* gene and TD phenotypes was reported in a study including 520 TD nuclear families (26). Also, rare, genic CNVs identified in 460 TD patients were enriched for HA pathway genes (27), supporting the potential involvement of the histamine pathway in TD etiology.

In the adult human central nervous system, the *HDC* gene is exclusively expressed in the soma and axon varicosities of HA neurons mostly originating from the tuberomammillary nucleus in the posterior hypothalamic region of the brain (30). The HDC homodimer converts l-histidine into histamine in the soma of HA neurons. Histamine-containing neuronal fibers are seen in many brain areas in rodents and human including cerebral cortex (56, 57). Therefore, loss-of-function mutations at the *HDC* gene will likely cause a lack of histamine in the widespread brain regions receiving HA innervation. In addition to serving as a neurotransmitter, histamine is a neuromodulator, inhibiting dopamine release by striatal dopaminergic neurons through binding to the H3 receptors expressed on these neurons in mice (58). Given the hypothesis that hyperactivity of nigrostriatal dopaminergic neurons is responsible for tic generation (12), it is reasonable that histamine dysregulation may contribute to TD.

Since HDC protein functions as a homodimer (59), individuals harboring the W317X mutation have approximately 25% residual HDC activity remaining compared to the healthy controls. Therefore, the *Hdc* knockout mice may recapitulate the behavioral outcomes caused by the W317X mutation in humans. As expected, the *Hdc* knockout mice show tic-like stereotypic behaviors after psychostimulant administration and reduced pre-pulse inhibition (28). Interestingly, the striatal dopamine level was increased in the *Hdc* knockout mice during the dark cycle, which could be decreased by administration of histamine in the knockout mice. Also, higher levels of dopamine D2 receptor occupancy were found in the basal ganglia of TD patients carrying the W317X mutations, the *Hdc* knockout mice and the *Hdc* heterozygous mice, indicating that the dopamine release in the basal ganglia of the brain might be disinhibited due to histamine depletion (28). Taken together, parallel studies in TD subjects and mice demonstrated that lack of histamine results in dopamine dysregulation, providing a potential mechanism for the proposed role of dopamine disruption in TD (12).

### Serotonin Pathway

#### Serotonin Transporter (*SLC6A4* or *SERT*)

Given the effectiveness of the SSRIs in reducing OCD symptoms, the *SERT* gene has been studied as candidate gene for OCD (60–64). Serotonin-transporter-linked polymorphic region (5-HTTLPR) polymorphisms and a gain-of-function missense mutation I425V have been associated with OCD (65–69). Sequence variants of the *SERT* gene were first associated with TD in a two-generation pedigree (24). In this family, the heterozygous “long” 5-HTTLPR variant and the I425V mutation perfectly segregated with TD individuals in a dominant pattern. The “long” 5-HTTLPR produces higher *SERT* mRNA level compared to the “short” 5-HTTLPR (70). The I425V mutation results in constitutive activation of the SERT protein whose activity is regulated by cGMP-dependent protein kinase (71). Therefore, carrying both “long” 5-HTTLPR and the I425V mutation is expected to have a synergistic effect that increases the expression of *SERT* mRNA and increases the amount of activated SERT protein.

In the mammalian central nervous system, the *SERT* gene is primarily expressed in the serotonergic neurons that originate from the raphe nucleus in the hindbrain and project widely to other parts of the nervous system, descending to the spinal cord and ascending to the forebrain (72). The human SERT protein is a transmembrane protein (73, 74). Cell surface expression of SERT protein can be regulated by SERT antagonists and substrates (75). The serotonergic axons can innervate and regulate other neurotransmission systems. For example, serotonin can facilitate or inhibit dopamine release in the striatum in a direct or indirect manner (76). Therefore, dysregulation of SERT expression on the plasma membrane may affect dopamine transmission (77). So far, no other sequence variants in the *SERT* gene have been associated with TD and no corresponding transgenic mice are available for *in vivo* studies.

#### Serotonin Receptor 1A (*HTR1A*) and Serotonin Receptor 2B (*HTR2B*)

In addition to the SERT gene, other serotonin pathway genes have been examined in TD patients. A missense mutation causing an amino acid change from arginine to leucine was identified in the serotonin receptor 1A gene (*HTR1A*) in one TD patient. However, the mutation was not predicted to change the receptor activity (78). Two novel non-synonymous missense variants and three known SNPs in the serotonin receptor 2B (*HTR2B*) gene were also found in 132 Caucasian and 128 Chinese Han TD individuals, though the associations were not statistically significant (79).

There are currently 14 known serotonin receptors and these are categorized into seven classes (80, 81). The *HTR1A* and *HTR2B*, both of which are G protein-coupled receptors, belong to class I and class II, respectively (81). The HTR1A receptor is highly expressed in the brain. Lower brain expression of the HTR1A receptor has been associated with mood disorders in humans. *Htr1a* knockout mice exhibit depression- and anxiety-like behaviors and have been used for antidepressant drug screening (82, 83). The HTR1A receptors are located at both pre-synaptic and post-synaptic neurons in the CNS. Activation of the pre-synaptic HTR1A receptors on the serotonergic neurons leads to inhibition of serotonergic neuron firing and reduced serotonin release whereas activation of the post-synaptic HTR1A can modulate the release of other neurotransmitters (84). Compared to the HTR1A receptor, the role that HTR2B plays in the CNS is not well understood. However, there is evidence suggesting that HTR2B may regulate SERT activity by phosphorylating SERT protein (85). In mice, the HTR2B receptor may be involved in modulating serotonin release from the serotonergic neurons. (86). Taken together, dysfunction of the HTR1A or the HTR2B receptor might lead to abnormal serotonin release in the CNS.

### Glutamate Pathway

#### Excitatory Amino Acid Transporter 1 (*SLC1A3* or *EAAT1*)

Altered cortico-striatal-thalamo-cortical (CSTC) circuitry is believed to provide the neurobiological basis for TD (7, 87). Glutamate is the major excitatory neurotransmitter in CSTS circuitry. A missense mutation (E219D) in the glutamate transporter gene (*SLC1A3*) was associated with TD in a case-control study (88). In the same study, cells transfected with the E219D mutant glutamate transporter gene exhibited increased glutamate uptake activity compared to cells transfected with the wild-type gene. The proposed mechanism for the increased glutamate uptake activity was elevated glutamate transporter expression at the plasma membrane due to the E219D mutation. However, whether TD might be associated with hypo- or hyper-glutamate activity is still controversial.

One of the five subtypes of glutamate transporters, EAAT1, is responsible for the reuptake of the excitatory neurotransmitter, l-Glutamate, from the synapses back into cells. Dysfunction of glutamate transporters may lead to imbalanced extracellular glutamate levels, further affecting downstream glutamate neurotransmission or causing glutamate excitotoxicity to neurons (89, 90). EAAT1 is primarily expressed in glial cells. Regionally, EAAT1 proteins are found in neocortex, striatum, cerebellum, and spinal cord (91). *Eaat1* knockout mice showed hyperactivity and reduced acoustic startle response compared with the wild-type mice (92) but did not exhibit the altered prepulse inhibition behavior, which has been found in TD patients (93). No *Eaat1* gain-of-function mutant mice are available to test the “hypo-glutamate activity” hypothesis in TD.

### Synaptic Signal Transduction and Cell-Adhesion Pathways

#### Neuroligin 4, X Linked (*NLGN4* or *NLGN4X*)

Mutations in the neuroligin (NLGN) family members have been identified in patients with neuropsychiatric disorders such as ASD (94–97) and schizophrenia (98). A small deletion in the *NLGN4* gene was detected in a mother and her two sons (99), one of whom was diagnosed with autism while the other was diagnosed with TD and ADHD. Their mother, who also carried the deletion, had a learning disability, depression, and anxiety. The deletion spanned exon 4, 5, and 6 of the *NLGN4* gene, resulting in a truncated protein. No other known genes were affected by the deletion.

Neuroligins are cell adhesion molecules located on the plasma membrane of the pre-synaptic and post-synaptic neurons. By interacting with neurexins, another family of cell adhesion proteins, NLGNs modulate proper signal transmission between pre-synaptic and post-synaptic neurons (100).Reduced expression of NLGNs in cultured neurons or mice cause deficits in synaptic maturation and plasticity (101, 102). *Nlgn4* knockout mice have been studied at both the behavioral and cellular levels. Because *Nlgn4* gene disruptions had been associated with ASD, the *Nlgn4* knockout mice were tested for ASD-like behaviors. As expected, *Nlgn4* knockout mice exhibited deficits in social interactions and reduced ultrasonic vocalizations compared to the wild-type mice (103). In another study, the *Nlgn4* knockout mice displayed reduced neural network response upon stimulation in both excitatory and inhibitory circuits (104). More interestingly, the *Nlgn4* knockout mice also showed stereotypic repetitive behaviors and increased obsessive compulsive like behaviors (105), supporting the possibility that disruption of the *NLGN4* gene might play a role in TD or related disorders.

#### Cadherin 2, Type1, N-Cadherin (*CDH2*)

Cadherin 2 (CDH2), also known as N-Cadherin, is another cell adhesion protein that has been associated with TD. CDH2 participates in neuron–neuron communication and in synaptogenesis. In a recent study, exons of the *CDH2* gene were sequenced in 160 OCD probands and 160 controls (106). Four variants in the *CDH2* gene were identified in subjects with OCD or TD. Two mutations were of particular interest: the N706S and the N845S variants. N706S is a rare and novel mutation close to the predicted proteolytic cleavage site of the CDH2 protein while the N845S variant is located at the β-catenin interacting region. Both mutations reduced the CDH2 protein level by more than 50% in transfected HEK293 cells (106), suggesting possible adhesion deficits in cells.

Cadherin 2 is a calcium-dependent cell–cell adhesion glycoprotein. CDH2 primarily mediates neurite outgrowth and axon guidance of neurons on myotubes (107) and on the surface of astrocytes (108, 109). The cytosolic domain of the CDH2 protein forms complexes with catenin proteins (110), and these complexes regulate synaptogenesis in both pre- and post-synaptic neurons (111, 112). Additionally, the cleaved C-terminal domain of the CDH2 is a repressor of CBP/CREB-mediated transcription whose target genes are critical in neural development and plasticity (113). The complete knockout of the *Cdh2* gene is an embryonic lethal in mice whereas *Cdh2* heterozygous null mice do not exhibit obvious morphological defects during development (114). Conditional knockout of the *Cdh2* gene in the cerebral cortex of mice caused disrupted cortical structure (115). Thus far, behavioral analyses in the *Cdh2*+*/*− mice or *Cdh2* conditional knockout mice have not been conducted.

#### Contactin-Associated Protein-Like 2 (*CNTNAP2/CASPR2*)

Variants in the *CNTNAP2* gene have been associated with ASD in several family based studies (116–119). Also, putative deleterious mutations were found in the *CNTNAP2* gene in ASD patients (120). In one family, an insertion on chromosome 7p35–p36, disrupting intron 8 of the *CNTNAP2* gene, was shared by a father and his two children, all three of whom were diagnosed with TD (121). However, a translocation disrupting intron 11 of *CNTNAP2* did not cause TD phenotypes in another three generation family (122).

CNTNAP2, a transmembrane protein, belongs to the neurexin superfamily and is highly expressed in neurons and localized to the axonal membrane of the juxtaparanodal region. The CNTNAP2 protein interacts with clustered Shaker-type potassium channels and plays an important role in the axon-glia septate-like junction (123). It is required for normal action potential propagation along myelinated axons of the neurons (124), and it has been hypothesized that malfunctions of the CNTNAP2 protein leads to deficits of electric signal transduction between neurons (121, 125, 126).

Behavioral assessments of *Cntnap2* knockout mice led to stereotypic motor movements (127). Interestingly, a reduced number of GABAergic interneurons were reported in the striatum of the *Cntnap2* knockout mice, which is consistent with previous post-mortem studies showing a reduction in the number of striatal GABAergic interneurons in TD patients (9, 21). Therefore, understanding the molecular mechanism of neuronal loss caused by disruption of CNTNAP2 may help to pinpoint biological pathways altered in TD.

#### Dipeptidyl-Peptidase 6

A heterozygous microdeletion at the first exon of the *DPP6* gene was identified in a boy with TD as well as the boy’s father and paternal uncle both of whom were diagnosed with tic disorder and ADHD (128). The microdeletion led to decreased *DPP6* mRNA level in the boy’s blood cells. *DPP6* has also been associated with other neuropsychiatric disorders such as ASD (95, 129) and schizophrenia (130).

Dipeptidyl-peptidase 6 (DPP6) is a transmembrane protein belonging to a family of serine proteases. However, DPP6 does not have protease activity (131). DPP6’s expression is enriched in the brain and different isoforms have different distributions in the brain. (132). DPP6 proteins interact with A-type voltage-gated potassium channels, specifically the Kv4 subunit (133, 134). The A-type potassium channels participate in the modulation of dendritic signal transmission (135, 136). Moreover, the A-type potassium channel was reported to control the tonic dopamine release by substantia nigra dopaminergic neurons (137). Even though DPP6 has no peptidase activity, it may have novel functions and play essential roles in Kv4 intracellular trafficking, membrane expression, and proper function of the A-type potassium channels (138). *Dpp6* knockout mice show abnormal synaptogenesis (139), and knocking down the *Dpp6* gene specifically in the mouse brain caused impaired learning and memory abilities (140).

### Mitochondria Functions

#### Inner Mitochondrial Membrane Peptidase 2 Like

A familial translocation segregating with TD or tics (141) and a *de novo* duplication in a TD patient with other developmental and mental phenotypes implicated *IMMP2L* as a possible TD candidate gene (142). This was the first mitochondria-related gene in TD. Subsequently, a cryptic deletion eliminating exons 1–3 of the *IMMP2L* gene and 21 other genes was identified in a TD patient with learning and speech difficulties (143). Also, a case-control study of copy number variations reported intragenic deletions at the *IMMP2L* gene in seven TD patients (144). Among the seven, three deletions at intron 3 led to production of a shorter *IMMP2L* mRNA transcript due to alternative splicing. In the same study, the expression of both the short and the long transcripts was examined by reverse transcription PCR in 19 regions of the human brain. While the long transcript was ubiquitously expressed in all 19 brain regions, the short transcript was selectively expressed at a lower level in only 10 brain regions, suggesting that the short transcript might have a more specific role in the human central nervous system (144).

The human inner mitochondrial membrane peptidase 2 like (IMMP2L) protein is one of the catalytic subunits of the inner mitochondrial membrane peptidase (IMP) complex (145, 146). The IMP complex participates in the cleavage of the inner mitochondrial membrane targeting signal sequence from its protein substrates, allowing maturation of the substrates. Loss of any subunit will cause the decomposition of the whole complex (147). Expression and function of the mammalian IMMP2L protein have been studied in animal models and human tissues. *Immp2l* knockout mice exhibit mitochondrial dysfunction, increased ischemic brain damage, and infertility (148, 149).The human *IMMP2L* mRNAs are ubiquitously expressed in various tissues except for adult liver and lungs. Unlike other TD associated genes, there is no enriched *IMMP2L* mRNA expression in the brain compared to other tissues (142). However, linking mitochondrial dysfunction to TD might lead to further speculation about the varied etiology of TD.

#### Mitochondrial Ribosomal Protein L3

Whole exome sequencing of a multiplex TD family showed three rare novel non-synonymous mutations in the *MRPL3*, *DNAJC13*, and the *OFCC1* genes (150). The three variants were not found in controls or dbSNPs/1000 Genomes databases. However, a targeted-sequencing study of the same three variants in Han Chinese TD patients did not replicate these findings (151).

Mitochondrial ribosomal protein L3 (MRPL3) is a mitochondrial ribosome protein involved in mitochondrial protein translation (152). Compound heterozygous mutations in the *MRPL3* gene were associated with mitochondrial respiratory chain deficiency in a pedigree of French origin (153), but no psychiatric diseases were reported.

### Genes Associated with Other Diseases

#### DnaJ (Hsp40) Homolog, Subfamily C, Member 13 (*DNAJC13*)

A missense variant (A2057S) in the *DNAJC13* gene was found to segregate with TD or chronic tic disorder (CTD) in a multiplex pedigree (150). Subsequently, a novel missense mutation Asn855Ser in the *DNAJC13* gene was found in a multi-generation family with PD and in an additional four other PD patients (154). Human DNAJC13 is a membrane-associated protein involved in endocytosis, specifically in the process of early endosome-mediated membrane trafficking (155, 156). Knocking down the *DNAJC13* gene in mammalian cells did not cause obvious dysfunction of endocytosis. However, introducing a C-terminus truncated mutant DNAJC13 protein into the cells did affect the normal distribution and formation of early endosomes (156). No *DNAJC13* knockout animal model is available.

#### Orofacial Cleft 1 Candidate 1

After an initial study suggesting that the *OFCC1* gene led to orofacial clefts (157), it was later linked to schizophrenia (158). Recently, a genome-wide association study (GWAS) of OCD found a significant association with *OFCC1* (159). Sequence variants in *OFCC1* have been found in patients with neurodevelopmental or neuropsychiatric disorders: a missense mutation (R129G) segregating with TD and CTD in a multiplex family (150) and a non-sense and a missense mutation were found in a single autism family (160).

The function of the orofacial cleft 1 candidate 1 (OFCC1) protein is unclear but one study suggested that the OFCC1 protein was an interacting partner and methylation substrate of protein arginine methyltransferase 1 (161). However, *Ofcc1* knockout mice did not show any behavioral abnormalities (158).

#### Hypocretin (Orexin) Receptor 2 (*HCRTR2/OX2R*)

The coding regions of the orexin-1/hypocretin-1 (*OX1R*) receptor gene, the orexin-2/hypocretin-2 (*OX2R*) gene, and the prepro-orexin gene were examined in patients with Excessive Daytime Sleepiness, TD, or ADHD and healthy controls (162). A C29T nucleotide change in the *OX2R* gene producing a Pro10Ser amino acid substitution was detected in only one TD patient with comorbid ADHD. The Pro10Ser variant reduces responsiveness of the Orexin2 receptor to its ligands, Orexin-A, and Orexin-B. The Orexin receptor 2 is a G protein-coupled receptor that participates the regulation of feeding and sleep-wakefulness in mammalian brains (163). *Ox2r* knockout mice do not show any tic-like behaviors (164).

## Functional Analyses of Genes Disrupted in TD

As indicated in the previous section, evidence for TD susceptibility genes exists. The mutations discussed were found in only a small number of individuals with TD, and replication remains elusive. This lack of replication may be due to the extreme locus heterogeneity, similar to what has been found in ASD (165). What evidence beyond stronger statistical association might help establish their potential role in TD pathogenesis? Some of these genes are found within neurobiological pathways that are presumed to be disrupted in TD (e.g., neural signal transmission/modulation) while others are found in novel pathways. Hence, the evidence that these genes are true susceptibility genes remains insufficient. Consequently, more convincing functional studies are needed to determine how variants in these genes could contribute to TD. In this next section, we review the *in vitro* and *in vivo* functional studies and techniques (beyond the knock out experiments referred to above) that will likely be useful to evaluate the consequences of mutations found in these presumptive TD susceptibility genes and for discovery of cellular and molecular phenotypes in disease.

### Transgenic Mammalian Cell Lines

Functional studies using neuronal cells from individuals with TD can provide insights into the molecular basis of TD and potentially help to clarify the biological pathways altered in TD. However, one of the difficulties is the inability to obtain relevant biomaterials (e.g., neurons or neural stem cells) from affected individuals. Since TD is not a lethal disorder, there is very limited access to neural tissue from individuals with TD, particularly from individuals with a known causal variant.

Transgenic human non-neuronal cell lines have been used to characterize the cellular and molecular phenotypes resulting from specific mutations. Typically, plasmids carrying a wild type or a mutant gene of interest are delivered into the cell lines. Once the protein products of the transgene are expressed in the cells, assays are developed to evaluate the functional consequences of the mutant proteins. For example, in the *CDH2* gene, both the wild type and mutant *CDH2* genes were cloned into expression plasmids and subsequently delivered into human embryonic kidney (HEK293) cells and reduced expression of mutant CDH2 proteins was reported (106). While easily done, functional studies in non-neuronal cell lines are suboptimal for a variety of reasons. During transgenesis, the gene of interest is often transiently overexpressed or is controlled by a conditional and/or inducible gene expression system (166). Therefore, the level of transgene expression might not faithfully represent the endogenous gene expression level. Also, the expression levels of many genes are tissue-specific (167). This is particularly relevant for genes with multiple transcript isoforms where the isoform expression pattern in transgenic cell lines may not be comparable to patterns in neurons. Furthermore, because transgenic cells would not be expected to have the same gene expression profile as neurons, they may not provide relevant cellular environment for the transgene to execute its genuine neuronal function(s). Neuronal samples from TD patients are therefore preferred for *in vitro* functional studies but these are very difficult if not impossible to obtain. A recent technological advance, induced pluripotent stem cells (iPSCs), now makes functional studies of neuronal samples with a known causal variant possible.

### Induced Pluripotent Stem Cells

#### Use of iPSC-Derived Neuronal Cells to Model Neuropsychiatric and Neurodevelopmental Disorders

The relatively new reprograming technique that converts human somatic cells into iPSCs allows researchers to model diseases *in vitro* using patient-derived cells. Since the first generation of iPSCs from mouse fibroblasts (168), the ability to produce differentiated cells from iPSCs has been intensively studied and improved. Recently, iPSC neuronal differentiation has become routine (169). Generating patient-specific neurons carrying mutations in disease candidate genes is invaluable for researchers who wish to study the cell-autonomous effects of mutations and to understand the cellular basis of neurological and neuropsychiatric disorders. To date, no study using iPSC-derived neurons to model TD has been published. However, mutations associated with other neuropsychiatric disorders have been studied in iPSC-derived neurons, and cellular abnormalities have been demonstrated (170–174). For example, Rett syndrome is a neurodevelopmental disorder occurring mainly in females characterized by mental retardation. Loss-of-function mutations in the methyl-CpG binding protein 2 (*MeCP2*) gene were reported in the majority of Rett Syndrome cases (170). Therefore, neurons with functional null mutation in the *MeCP2* gene were generated from the iPSCs of an individual with Rett Syndrome. In culture, the neurons showed smaller soma size (171). Similarly, iPSC-derived neurons have been used to understand ASD. In an individual with ASD, a balanced translocation spanning the transient receptor potential 6 channel (*TRPC6*) gene was identified. Neurons derived from the iPSCs exhibited decreased *TRPC6* expression, altered morphology and reduced Ca^2+^ influx (172). In schizophrenia, the *DISC1* gene has been considered an important risk factor (175) and in iPSC-derived *DISC1* mutant neurons, pre-synaptic vesicle release was impaired (173).

Unfortunately, *in vitro* neural differentiation from iPSCs yields mixed populations of neurons rather than a homogenous population. For diseases with clear neuropathology, pure cultures of the specific neuron types involved in the disorder are preferred in order to recapitulate disease-related cellular phenotypes. For instance, PD is characterized by loss of substantia nigra dopamine neurons (176). Cultures containing a high percentage of dopaminergic neurons were generated from PD patients who carry monogenic mutations (177–180), and defects in cellular functions such as autophagy, mitophagy, oxidative stress response, and dopamine release were found in these neurons. In patients with amyotrophic lateral sclerosis (ALS), motor neurons degenerate. Therefore, motor neurons were derived from iPSCs of ALS patients carrying known casual mutations (174, 181). As hypothesized, the mutant motor neurons exhibited neurite degeneration (181).

#### Use of Single-Cell Analysis to Overcome Culture Heterogeneity

One method to overcome cell culture heterogeneity is to analyze single cells, all of the same type. Looking at the transcriptome of single cells using microarray or RNA-seq analysis holds promise of detecting gene dysregulation in particular populations of neurons, which might not be identified by analyzing heterogeneous mixtures of cells. For instance, single-cell gene expression analysis of iPSC-derived dopamine neurons from PD patients with a LRKK2 mutation unveiled dysregulation of oxidative stress genes in mutant dopamine neurons (182). In another study, neurons were generated from the iPSCs of Timothy Syndrome patients with a mutation in the *CACNA1C* gene. Single cells were taken from the culture containing mixed neuron populations, and gene expression was analyzed by microarray. As a result, the distribution of neuron subtypes was altered in the Timothy Syndrome neuronal culture compared to the control cells (183). Compared to microarray, RNA-seq is gaining greater popularity for analyzing the transcriptome of single cells due to its ability to unbiasedly detect any transcript in cells within a broader dynamic range of expression. Generally, there are four important steps to achieve single-cell RNA-seq: (1) single-cell isolation, (2) RNA capture, (3) cDNA synthesis and, (4) next generation sequencing. The microfluidic system is becoming popular for single-cell RNA-seq because it can isolate single cells, lyse the cells, purify RNA, synthesize cDNA or even conduct gene expression analysis all in one run (184, 185).

#### Use of iPSC-Derived Cerebral Organoids to Model Neuropsychiatric and Neurodevelopmental Disorders

In contrast to PD or ALS, the neuropathology of many neurodevelopmental and neuropsychiatric disorders, such as TD or ASD, is unclear or is heterogeneous (18). As described above, mutations associated with TD indicate dysregulations of various neurotransmitter pathways or of neural circuits involving multiple brain regions. Hence, studying specific type of neurons may not help to explain the pathogenesis of TD. The recently developed “cerebral organoid” cellular system enables the differentiation of iPSCs into a three-dimensional miniature organ in a bioreactor, with minimum external interferences (186). Such self-organized spherical structures resemble the human brain at very early stages of development. In comparison to monolayer neuronal cultures, the cerebral organoids contain more diverse neuronal populations that define distinct brain regions. Also, within the cerebral organoid, neuronal migration and human-specific brain structures (e.g., the outer subventricular zone) were observed (187). Therefore, the cerebral organoid has been used to model neurodevelopmental diseases. For microcephaly, premature neural differentiation was recapitulated in organoids derived from microcephaly patients’ iPSCs (187). In cases of idiopathic ASD, overproduction of GABAergic inhibitory neurons in patient-derived cerebral organoids was reported (188). At the molecular level, the cellular phenotype was explained by overexpression of the transcription factor FOXG1. However, use of cerebral organoids to model neurodevelopmental diseases has limitations. The various “brain regions” in the organoids are fairly disorganized. Therefore, the cerebral organoid would not be suitable to study neural circuits. Furthermore, neuronal cells within the organoids are mostly neural progenitor cells, and their differentiation is restricted by limited growth of the organoids, which in turn is probably due to the lack of internal nutrient and oxygen supply. More importantly, each organoid is “unique” because the self-organization process is random and is not controlled by external factors. This “uniqueness” will generate variation among organoids, which may mask phenotypic differences between normal and patient-derived cells.

#### Use of Genome Editing to Generate Isogenic Control iPSC Lines

One challenge in identifying the phenotypic effects of a given mutation in iPSC-derived neurons is finding an appropriate control sample. While age, gender, and ethnicity-matched control samples with the wild-type allele are typically available, they are not matched for all of the other common genomic variants. Failure to control for such variability in genetic background can lead to spurious results. The recent technological advance of highly specific genome editing now allows the production of more comparable isogenic controls for functional studies. Several genome editing systems, such as zinc finger nuclease (189, 190), TALEN, and CRISPR-Cas9 (191, 192), are able to reverse the mutation to wild type at one genomic locus at a time in iPSCs. Comparing neuronal cells generated from mutant iPSCs and their edited, isogenic control neuronal cells with the mutation removed allows identification of molecular and cellular changes that are due only to the mutation (180). However, “off target” mutations at unrelated loci inadvertently introduced by editing remain a potentially important technological hurdle (193).

### Gene Expression and Gene Network Analyses

The major goal of genomic sequencing of patients with neurodevelopmental and neuropsychiatric disorders is to identify disease-associated mutations. Once such genes are found, systematic approaches including genome-wide gene expression analysis and gene network analyses can be used to implicate common biological pathways altered in patients with different mutant genes.

#### Gene Expression Analysis

Gene expression analysis, primarily through the RNA-seq approach, aims to quantify transcript level of target genes or of the whole transcriptome in biological samples from patients and healthy controls to identify genes dysregulated in human diseases (194). For neurodevelopmental disorders, post-mortem brain samples are often used for the transcriptomic analyses. The first transcriptomic analysis of TD patients’ post-mortem striatum samples revealed that interneuron disruption might be involved in the pathophysiology of TD (34). However, to evaluate particular mutations, post-mortem samples meeting specific research criteria are usually difficult or virtually impossible to obtain. With the emergence of somatic cell reprograming techniques, iPSC-derived neurons with and without a putative disease-causing mutation can be produced *in vitro* (195). The transcriptomes of these iPSC-derived neurons can be compared by microarray or RNA sequencing (RNA-seq) (172, 173). To further dissect the cellular phenotype at single neuron level and to detect abnormalities only shown in particular populations of neurons, single-cell transcriptome analysis can also be performed (182, 183). Multiple bioinformatic tools have been developed for RNA-seq data to detect differentially expressed genes (DEGs) from distinct cell types or under different experimental conditions (196–198). Among these RNA-seq analysis methods, none outperforms the others in all aspects. Selecting an optimal method for a study requires an understanding of the benefits and limitations of each method as well as the parameters of the study (196). Once the DEGs between experimental conditions are determined, gene and pathway annotation tools, gene and protein expression and interaction databases can help to explore the gene pathways underlying the disorder. Gene and pathway annotations tools such as IPA^1^, KEGG (199, 200), DAVID (201, 202), ConsensusPathDB (203) report biological pathways in which DEGs are enriched or reduced and take these into account to predict how these pathways might be affected. However, the data from which these tools were constructed come from non-neuronal samples which could lead to associations not found in neuronal tissues or failure to detect neuronal associations (204). In order to annotate neuronal gene expression in a temporal and spatial manner, human brain gene expression databases, for example, the Allen Human Brain Atlas (205), BrainSpan (206), GTEx (207) were built using microarray and RNA-seq data from post-mortem brain samples. Mapping DEGs identified in neuronal samples of patients with neurodevelopmental disorders to human brain gene expression databases revealed specific brain regions and neural developmental stages that were affected (34, 208). A more detailed human brain gene expression atlas that annotates gene expression at single cell level has been initiated by a group in Stanford University (209). Single-cell RNA-seq was used to analyze neurons from human adult and fetal cerebral cortex and it identified more diverse populations of neurons within the cortical region (209). Constructing a comprehensive human brain gene expression database at single neuron resolution is quite challenging due to limited access to healthy human brain samples and the high cost of single-cell RNA-seq. Therefore, collaborative work with standardized experimental protocols is required.

#### Gene Network Analysis

Differential gene expression from transcriptome analysis is sample-dependent and tissue-specific. In order to explore the etiology of complex neurodevelopmental disorders such as TD, disease-associated genes can be mapped to gene networks to visualize relationships between disease candidate genes and, further, to pinpoint annotated or novel pathways. The gene networks can be gene co-expression networks (205, 206), gene regulatory networks^2^, protein–protein interaction networks (210) or networks constructed with combined criteria (203, 211). For example, in ASD, disease-associated genes have been evaluated using spatiotemporal gene co-expression networks constructed from BrainSpan (206) and were found to be enriched in sub-networks that represent specific brain regions and time periods during human brain development (212). Furthermore, ASD-associated mutations identified by previous genetic studies were mapped to a “background network” which scores each pair of human genes based on very comprehensive information about every known human gene (211). Cell types and brain areas affected in ASD were implicated (213). With a growing number of mutations associated with TD, the same approaches could be utilized in TD.

### Animal Models

Another approach to the study of the functional effects of a specific mutation in a gene is to use animal models. Most often, a putative disease gene is knocked out or modified in the animal model. Then, the mutant and wild type animals from the same genetic background are compared. (Some such animal knock-out studies of putative TD genes have been described above). Conventional animal models are designed to study a small number of genes, usually one or two genes at a time. If, however, TD is caused by the combined effect of multiple variant genes, multi-transgenic animals, whose genomes are modified at multiple loci, would be required. Although more challenging, generation of such multi-transgenic animals can be achieved by genome editing as well. (214, 215).

In order to model TD in animals, the following criteria should be met: (1) the gene to be studied is strongly associated with the disease; (2) the gene and the neurological component phenotypes involved in TD are relatively well conserved between humans and the animal; (3) the gene is thought to have similar functions in both humans and the model animals; and (4) the disease phenotype can be experimentally characterized in the model animals by biochemical and/or behavioral approaches (216). One mouse model in TD that meets these criteria is *HDC*. As indicated previously, a rare dominant non-sense mutation W317X in *HDC* cosegregated with all TD individuals in a two generation pedigree. (25). Subsequently, a study using *HDC* knockout mice demonstrated behavioral and molecular abnormalities caused by the loss of the HDC activity in the brain (28). The knockout and heterozygous mice showed tic-like stereotypic movements after psychostimulant administration. Also, the striatal dopaminergic pathway was dysregulated due to the HDC deficiency. Specifically, the dysregulation of dopamine receptors in the basal ganglia region of the *HDC* knockout and heterozygous mice recapitulated the dysregulation of the same types of dopamine receptors in TD patients carrying the W317X mutation.

## Summary and Future Directions

Tourette’s disorder is likely caused by a complex multigenic inheritance pattern that includes locus and allelic heterogeneity of both common and rare variants that interact with environmental factors (14, 217, 218). Despite long-standing interest in the genetic contribution to TD, the overall genetic architecture of TD remains elusive. Some of the genes identified as causal of TD are involved in neurotransmitter pathways presumed to be altered in TD, while others are novel. In this respect, the genetics of TD may resemble that of other complex neuropsychiatric disorders. Indeed, there is evidence of some overlap with subsets of similar genes involved in multiple disorders. Furthermore, there also appears to be an increased rate of comorbidity between some such disorders, such as TD and ASD (27, 219). It may also be that current DSM-based psychiatric nosology does not sufficiently “carve nature at its joints,” and that other classification schemes, such as Research Domain Criteria (RDoC)(220–224), might reveal etiologically more coherent groupings of disorders or patients.

International consortiums including the Tourette International Collaborative Genetics (TIC Genetics), the Tourette Syndrome Association International Consortium for Genetics (TSAICG), the European Multicenter Tics in Children Studies (EMTICS), the European Society for the Study of Tourette Syndrome (ESSTS), the Tourette Syndrome Genetics The Southern and Eastern Europe initiative (TSGeneSEE), and sharing repositories (New Jersey Center for Tourette Syndrome Repository) (225, 226) have initiated large collaborations to collect many patient and family samples in an effort to understand the genetics of TD. Continued efforts in gene discovery from large open-access repositories are needed to find additional risk variants.

## Author Contributions

All authors wrote, reviewed, and critiqued the manuscript.

## Conflict of Interest Statement

The authors declare that the research was conducted in the absence of any commercial or financial relationships that could be construed as a potential conflict of interest. The reviewer Fotis Tsetsos and handling Editor Peristera Paschou declared their shared affiliation, and the handling Editor states that the process nevertheless met the standards of a fair and objective review.

## Footnotes

^1^www.qiagen.com/ingenuity

^2^http://www.braineac.org/

## References

[1] Robertson MM. Diagnosing Tourette syndrome: is it a common disorder? J Psychosom Res (2003) 55(1):3–6. 10.1016/S0022-3999(02)00580-9 12842225

[2] Scharf JM Miller LL Gauvin CA Alabiso J Mathews CA Ben-Shlomo Y. Population prevalence of Tourette syndrome: a systematic review and meta-analysis. Mov Disord (2015) 30(2):221–8. 10.1002/mds.26089 25487709

[3] Robertson MM. The Gilles de la Tourette syndrome: the current status. Arch Dis Child Educ Pract Ed (2012) 97(5):166–75. 10.1136/archdischild-2011-300585 22440810

[4] Shaw ZA Coffey BJ. Tics and tourette syndrome. Psychiatr Clin North Am (2014) 37(3):269–86. 10.1016/j.psc.2014.05.001 25150562

[5] Freeman RD Fast DK Burd L Kerbeshian J Robertson MM Sandor P. An international perspective on Tourette syndrome: selected findings from 3,500 individuals in 22 countries. Dev Med Child Neurol (2000) 42(7):436–47. 10.1017/S0012162200000839 10972415

[6] Paschou P Fernandez TV Sharp F Heiman GA Hoekstra PJ. Genetic susceptibility and neurotransmitters in Tourette syndrome. Int Rev Neurobiol (2013) 112:155–77. 10.1016/B978-0-12-411546-0.00006-8 24295621 PMC4471172

[7] Leckman JF Bloch MH Smith ME Larabi D Hampson M. Neurobiological substrates of Tourette’s disorder. J Child Adolesc Psychopharmacol (2010) 20(4):237–47. 10.1089/cap.2009.0118 20807062 PMC2958453

[8] Wang ZS Maia TV Marsh R Colibazzi T Gerber A Peterson BS. The neural circuits that generate tics in Tourette’s syndrome. Am J Psychiatry (2011) 168(12):1326–37. 10.1176/appi.ajp.2011.09111692 21955933 PMC4246702

[9] Kataoka Y Kalanithi PSA Grantz H Schwartz ML Saper C Leckman JF Decreased number of parvalbumin and cholinergic interneurons in the striatum of individuals with Tourette syndrome. J Comp Neurol (2010) 518(3):277–91. 10.1002/cne.22206 19941350 PMC2846837

[10] Priori A Giannicola G Rosa M Marceglia S Servello D Sassi M Deep brain electrophysiological recordings provide clues to the pathophysiology of Tourette syndrome. Neurosci Biobehav Rev (2013) 37(6):1063–8. 10.1016/j.neubiorev.2013.01.011 23333267

[11] Pogorelov V Xu MY Smith HR Buchanan GF Pittenger C. Corticostriatal interactions in the generation of tic-like behaviors after local striatal disinhibition. Exp Neurol (2015) 265:122–8. 10.1016/j.expneurol.2015.01.001 25597650 PMC4361636

[12] Buse J Schoenefeld K Munchau A Roessner V. Neuromodulation in Tourette syndrome: dopamine and beyond. Neurosci Biobehav Rev (2013) 37(6):1069–84. 10.1016/j.neubiorev.2012.10.004 23085211

[13] Surmeier DJ Ding J Day M Wang Z Shen W. D1 and D2 dopamine-receptor modulation of striatal glutamatergic signaling in striatal medium spiny neurons. Trends Neurosci (2007) 30(5):228–35. 10.1016/j.tins.2007.03.008 17408758

[14] Bloch M State M Pittenger C. Recent advances in Tourette syndrome. Curr Opin Neurol (2011) 24(2):119–25. 10.1097/WCO.0b013e328344648c 21386676 PMC4065550

[15] Roessner V Plessen KJ Rothenberger A Ludolph AG Rizzo R Skov L European clinical guidelines for Tourette syndrome and other tic disorders. Part II: pharmacological treatment. Eur Child Adolesc Psychiatry (2011) 20(4):173–96. 10.1007/s00787-011-0163-7 21445724 PMC3065650

[16] Minzer K Lee O Hong JJ Singer HS. Increased prefrontal D2 protein in Tourette syndrome: a postmortem analysis of frontal cortex and striatum. J Neurol Sci (2004) 219(1–2):55–61. 10.1016/j.jns.2003.12.006 15050438

[17] Yoon DY Gause CD Leckman JF Singer HS. Frontal dopaminergic abnormality in Tourette syndrome: a postmortem analysis. J Neurol Sci (2007) 255(1–2):50–6. 10.1016/j.jns.2007.01.069 17337006

[18] Felling RJ Singer HS. Neurobiology of tourette syndrome: current status and need for further investigation. J Neurosci (2011) 31(35):12387–95. 10.1523/JNEUROSCI.0150-11.2011 21880899 PMC6703258

[19] Anderson GM Pollak ES Chatterjee D Leckman JF Riddle MA Cohen DJ Brain monoamines and amino acids in Gilles de la Tourette’s syndrome: a preliminary study of subcortical regions. Arch Gen Psychiatry (1992) 49(7):584–6. 10.1001/archpsyc.1992.01820070078016 1378261

[20] Singer HS Morris C Grados M. Glutamatergic modulatory therapy for Tourette syndrome. Med Hypotheses (2010) 74(5):862–7. 10.1016/j.mehy.2009.11.028 20022434

[21] Kalanithi PS Zheng W Kataoka Y DiFiglia M Grantz H Saper CB Altered parvalbumin-positive neuron distribution in basal ganglia of individuals with Tourette syndrome. Proc Natl Acad Sci U S A (2005) 102(37):13307–12. 10.1073/pnas.0502624102 16131542 PMC1201574

[22] Pittenger C Bloch MH. Pharmacological treatment of obsessive-compulsive disorder. Psychiatr Clin North Am (2014) 37(3):375–91. 10.1016/j.psc.2014.05.006 25150568 PMC4143776

[23] Roessner V Schoenefeld K Buse J Bender S Ehrlich S Munchau A. Pharmacological treatment of tic disorders and Tourette syndrome. Neuropharmacology (2013) 68:143–9. 10.1016/j.neuropharm.2012.05.043 22728760

[24] Moya PR Wendland JR Rubenstein LM Timpano KR Heiman GA Tischfield JA Common and rare alleles of the serotonin transporter gene, SLC6A4, associated with Tourette’s disorder. Mov Disord (2013) 28(9):1263–70. 10.1002/mds.25460 23630162 PMC3766488

[25] Ercan-Sencicek AG Stillman AA Ghosh AK Bilguvar K O’Roak BJ Mason CE l-histidine decarboxylase and Tourette’s syndrome. N Engl J Med (2010) 362(20):1901–8. 10.1056/NEJMoa0907006 20445167 PMC2894694

[26] Karagiannidis I Dehning S Sandor P Tarnok Z Rizzo R Wolanczyk T Support of the histaminergic hypothesis in Tourette syndrome: association of the histamine decarboxylase gene in a large sample of families. J Med Genet (2013) 50(11):760–4. 10.1136/jmedgenet-2013-101637 23825391

[27] Fernandez TV Sanders SJ Yurkiewicz IR Ercan-Sencicek AG Kim YS Fishman DO Rare copy number variants in tourette syndrome disrupt genes in histaminergic pathways and overlap with autism. Biol Psychiatry (2012) 71(5):392–402. 10.1016/j.biopsych.2011.09.034 22169095 PMC3282144

[28] Castellan Baldan L Williams KA Gallezot JD Pogorelov V Rapanelli M Crowley M Histidine decarboxylase deficiency causes tourette syndrome: parallel findings in humans and mice. Neuron (2014) 81(1):77–90. 10.1016/j.neuron.2013.10.052 24411733 PMC3894588

[29] Parent M Wallman MJ Gagnon D Parent A. Serotonin innervation of basal ganglia in monkeys and humans. J Chem Neuroanat (2011) 41(4):256–65. 10.1016/j.jchemneu.2011.04.005 21664455

[30] Haas HL Sergeeva OA Selbach O Histamine in the nervous system. Physiol Rev (2008) 88(3):1183–241. 10.1152/physrev.00043.2007 18626069

[31] Di Giovanni G Di Matteo V Esposito E Serotonin-dopamine interaction: experimental evidence and therapeutic relevance. Preface. Prog Brain Res (2008) 172:ix 10.1016/S0079-6123(08)00931-X 18772024

[32] Frick LR Williams K Pittenger C. Microglial dysregulation in psychiatric disease. Clin Dev Immunol (2013) 2013:608654. 10.1155/2013/608654 23690824 PMC3652125

[33] Morer A Chae W Henegariu O Bothwell AL Leckman JF Kawikova I. Elevated expression of MCP-1, IL-2 and PTPR-N in basal ganglia of Tourette syndrome cases. Brain Behav Immun (2010) 24(7):1069–73. 10.1016/j.bbi.2010.02.007 20193755

[34] Lennington JB Coppola G Kataoka-Sasaki Y Fernandez TV Palejev D Li Y Transcriptome analysis of the human striatum in Tourette syndrome. Biol Psychiatry (2014). 10.1016/j.biopsych.2014.07.018 25199956 PMC4305353

[35] Sundaram SK Huq AM Wilson BJ Chugani HT. Tourette syndrome is associated with recurrent exonic copy number variants. Neurology (2010) 74(20):1583–90. 10.1212/WNL.0b013e3181e0f147 20427753 PMC2876824

[36] Nag A Bochukova EG Kremeyer B Campbell DD Muller H Valencia-Duarte AV CNV analysis in Tourette syndrome implicates large genomic rearrangements in COL8A1 and NRXN1. PLoS One (2013) 8(3):e59061. 10.1371/journal.pone.0059061 23533600 PMC3606459

[37] Bertelsen B Stefansson H Riff Jensen L Melchior L Mol Debes N Groth C Association of AADAC deletion and Gilles de la Tourette syndrome in a large European cohort. Biol Psychiatry (2015). 10.1016/j.biopsych.2015.08.027 26444075

[38] Abelson JF Kwan KY O’Roak BJ Baek DY Stillman AA Morgan TM Sequence variants in SLITRK1 are associated with Tourette’s syndrome. Science (2005) 310(5746):317–20. 10.1126/science.1116502 16224024

[39] Deng H Le WD Xie WJ Jankovic J. Examination of the SLITRK1 gene in Caucasian patients with Tourette syndrome. Acta Neurol Scand (2006) 114(6):400–2. 10.1111/j.1600-0404.2006.00706.x 17083340

[40] Verkerk AJ Cath DC van der Linde HC Both J Heutink P Breedveld G Genetic and clinical analysis of a large Dutch Gilles de la Tourette family. Mol Psychiatry (2006) 11(10):954–64. 10.1038/sj.mp.4001877 16894393

[41] Chou IC Wan L Liu SC Tsai CH Tsai FJ. Association of the slit and Trk-like 1 gene in Taiwanese patients with Tourette syndrome. Pediatr Neurol (2007) 37(6):404–6. 10.1016/j.pediatrneurol.2007.06.017 18021920

[42] Scharf JM Moorjani P Fagerness J Platko JV Illmann C Galloway B Lack of association between SLITRK1var321 and Tourette syndrome in a large family-based sample. Neurology (2008) 70(16 Pt 2):1495–6. 10.1212/01.wnl.0000296833.25484.bb 18413575 PMC4655203

[43] Zimprich A Hatala K Riederer F Stogmann E Aschauer HN Stamenkovic M. Sequence analysis of the complete SLITRK1 gene in Austrian patients with Tourette’s disorder. Psychiatr Genet (2008) 18(6):308–9. 10.1097/YPG.0b013e3283060f6f 19018236

[44] Miranda DM Wigg K Kabia EM Feng Y Sandor P Barr CL. Association of SLITRK1 to Gilles de la Tourette syndrome. Am J Med Genet B Neuropsychiatr Genet (2009) 150B(4):483–6. 10.1002/ajmg.b.30840 18698576

[45] O’Roak BJ Morgan TM Fishman DO Saus E Alonso P Gratacos M Additional support for the association of SLITRK1 var321 and Tourette syndrome. Mol Psychiatry (2010) 15(5):447–50. 10.1038/mp.2009.105 20351724 PMC3292207

[46] Yasmeen S Melchior L Bertelsen B Skov L Mol Debes N Tumer Z. Sequence analysis of SLITRK1 for var321 in Danish patients with Tourette syndrome and review of the literature. Psychiatr Genet (2013) 23(3):130–3. 10.1097/YPG.0b013e328360c880 23528612

[47] Inai A Tochigi M Kuwabara H Nishimura F Kato K Eriguchi Y Analysis of SLITRK1 in Japanese patients with Tourette syndrome using a next-generation sequencer. Psychiatr Genet (2015) 25(6):256–8. 10.1097/YPG.0000000000000104 26317387

[48] Keen-Kim D Mathews CA Reus VI Lowe TL Herrera LD Budman CL Overrepresentation of rare variants in a specific ethnic group may confuse interpretation of association analyses. Hum Mol Genet (2006) 15(22):3324–8. 10.1093/hmg/ddl408 17035247

[49] Karagiannidis I Rizzo R Tarnok Z Wolanczyk T Hebebrand J Nothen MM Replication of association between a SLITRK1 haplotype and Tourette syndrome in a large sample of families. Mol Psychiatry (2012) 17(7):665–8. 10.1038/mp.2011.151 22083730

[50] Proenca CC Gao KP Shmelkov SV Rafii S Lee FS. Slitrks as emerging candidate genes involved in neuropsychiatric disorders. Trends Neurosci (2011) 34(3):143–53. 10.1016/j.tins.2011.01.001 21315458 PMC3051006

[51] Aruga J Mikoshiba K. Identification and characterization of Slitrk, a novel neuronal transmembrane protein family controlling neurite outgrowth. Mol Cell Neurosci (2003) 24(1):117–29. 10.1016/S1044-7431(03)00129-5 14550773

[52] Yim YS Kwon Y Nam J Yoon HI Lee K Kim DG Slitrks control excitatory and inhibitory synapse formation with LAR receptor protein tyrosine phosphatases. Proc Natl Acad Sci U S A (2013) 110(10):4057–62. 10.1073/pnas.1209881110 23345436 PMC3593915

[53] Um JW Kim KH Park BS Choi Y Kim D Kim CY Structural basis for LAR-RPTP/Slitrk complex-mediated synaptic adhesion. Nat Commun (2014) 5:5423. 10.1038/ncomms6423 25394468

[54] Katayama K Yamada K Ornthanalai VG Inoue T Ota M Murphy NP Slitrk1-deficient mice display elevated anxiety-like behavior and noradrenergic abnormalities. Mol Psychiatry (2010) 15(2):177–84. 10.1038/mp.2008.97 18794888

[55] Lei J Deng X Zhang J Su L Xu H Liang H Mutation screening of the HDC gene in Chinese Han patients with Tourette syndrome. Am J Med Genet B Neuropsychiatr Genet (2012) 159B(1):72–6. 10.1002/ajmg.b.32003 22095709

[56] Panula P Airaksinen MS Pirvola U Kotilainen E. A histamine-containing neuronal system in human brain. Neuroscience (1990) 34(1):127–32. 10.1016/0306-4522(90)90307-P 2325846

[57] Watanabe T Taguchi Y Shiosaka S Tanaka J Kubota H Terano Y Distribution of the histaminergic neuron system in the central nervous system of rats; a fluorescent immunohistochemical analysis with histidine decarboxylase as a marker. Brain Res (1984) 295(1):13–25. 10.1016/0006-8993(84)90811-4 6713171

[58] Schlicker E Fink K Detzner M Gothert M. Histamine inhibits dopamine release in the mouse striatum via presynaptic H3 receptors. J Neural Transm Gen Sect (1993) 93(1):1–10. 10.1007/BF01244933 8396945

[59] Komori H Nitta Y Ueno H Higuchi Y. Structural study reveals that Ser-354 determines substrate specificity on human histidine decarboxylase. J Biol Chem (2012) 287(34):29175–83. 10.1074/jbc.M112.381897 22767596 PMC3436558

[60] Zohar J Insel TR. Drug treatment of obsessive-compulsive disorder. J Affect Disord (1987) 13(2):193–202. 10.1016/0165-0327(87)90023-1 2960711

[61] Lieberman J. Evidence for a biological hypothesis of obsessive-compulsive disorder. Neuropsychobiology (1984) 11(1):14–21. 10.1159/000118043 6204247

[62] Zohar J Insel TR Zohar-Kadouch RC Hill JL Murphy DL. Serotonergic responsivity in obsessive-compulsive disorder. Effects of chronic clomipramine treatment. Arch Gen Psychiatry (1988) 45(2):167–72. 10.1001/archpsyc.1988.01800260081011 3276283

[63] Billett EA Richter MA King N Heils A Lesch KP Kennedy JL. Obsessive compulsive disorder, response to serotonin reuptake inhibitors and the serotonin transporter gene. Mol Psychiatry (1997) 2(5):403–6. 10.1038/sj.mp.4000257 9322235

[64] McDougle CJ Epperson CN Price LH Gelernter J. Evidence for linkage disequilibrium between serotonin transporter protein gene (SLC6A4) and obsessive compulsive disorder. Mol Psychiatry (1998) 3(3):270–3. 10.1038/sj.mp.4000391 9672904

[65] Bengel D Greenberg BD Cora-Locatelli G Altemus M Heils A Li Q Association of the serotonin transporter promoter regulatory region polymorphism and obsessive-compulsive disorder. Mol Psychiatry (1999) 4(5):463–6. 10.1038/sj.mp.4000550 10523819

[66] Di Bella D Erzegovesi S Cavallini MC Bellodi L. Obsessive-compulsive disorder, 5-HTTLPR polymorphism and treatment response. Pharmacogenomics J (2002) 2(3):176–81. 10.1038/sj.tpj.6500090 12082589

[67] Ozaki N Goldman D Kaye WH Plotnicov K Greenberg BD Lappalainen J Serotonin transporter missense mutation associated with a complex neuropsychiatric phenotype. Mol Psychiatry (2003) 8(11):933–6. 10.1038/sj.mp.4001415 14593431

[68] Wendland JR Moya PR Kruse MR Ren-Patterson RF Jensen CL Timpano KR A novel, putative gain-of-function haplotype at SLC6A4 associates with obsessive-compulsive disorder. Hum Mol Genet (2008) 17(5):717–23. 10.1093/hmg/ddm343 18055562

[69] Voyiaziakis E Evgrafov O Li D Yoon HJ Tabares P Samuels J Association of SLC6A4 variants with obsessive-compulsive disorder in a large multicenter US family study. Mol Psychiatry (2011) 16(1):108–20. 10.1038/mp.2009.100 19806148 PMC6528171

[70] Hu XZ Lipsky RH Zhu G Akhtar LA Taubman J Greenberg BD Serotonin transporter promoter gain-of-function genotypes are linked to obsessive-compulsive disorder. Am J Hum Genet (2006) 78(5):815–26. 10.1086/503850 16642437 PMC1474042

[71] Kilic F Murphy DL Rudnick G. A human serotonin transporter mutation causes constitutive activation of transport activity. Mol Pharmacol (2003) 64(2):440–6. 10.1124/mol.64.2.440 12869649

[72] Jacobs BL Azmitia EC Structure and function of the brain serotonin system. Physiol Rev (1992) 72(1):165–229. 1731370 10.1152/physrev.1992.72.1.165

[73] Blakely RD De Felice LJ Hartzell HC. Molecular physiology of norepinephrine and serotonin transporters. J Exp Biol (1994) 196:263–81. 7823027 10.1242/jeb.196.1.263

[74] Lesch KP Wolozin BL Estler HC Murphy DL Riederer P. Isolation of a cDNA encoding the human brain serotonin transporter. J Neural Transm Gen Sect (1993) 91(1):67–72. 10.1007/BF01244919 8452685

[75] Kittler K Lau T Schloss P. Antagonists and substrates differentially regulate serotonin transporter cell surface expression in serotonergic neurons. Eur J Pharmacol (2010) 629(1–3):63–7. 10.1016/j.ejphar.2009.12.010 20006597

[76] Esposito E Di Matteo V Di Giovanni G. Serotonin-dopamine interaction: an overview. Prog Brain Res (2008) 172:3–6. 10.1016/S0079-6123(08)00901-1 18772025

[77] Daw ND Kakade S Dayan P. Opponent interactions between serotonin and dopamine. Neural Netw (2002) 15(4–6):603–16. 10.1016/S0893-6080(02)00052-7 12371515

[78] Lam S Shen Y Nguyen T Messier TL Brann M Comings D A serotonin receptor gene (5HT1A) variant found in a Tourette’s syndrome patient. Biochem Biophys Res Commun (1996) 219(3):853–8. 10.1006/bbrc.1996.0322 8645269

[79] Guo Y Deng X Jankovic J Su L Zhang J Le W Mutation screening of the HTR2B gene in patients with Tourette syndrome. Neurosci Lett (2012) 526(2):150–3. 10.1016/j.neulet.2012.08.013 22917605

[80] Hannon J Hoyer D. Molecular biology of 5-HT receptors. Behav Brain Res (2008) 195(1):198–213. 10.1016/j.bbr.2008.03.020 18571247

[81] McCorvy JD Roth BL. Structure and function of serotonin G protein-coupled receptors. Pharmacol Ther (2015) 150:129–42. 10.1016/j.pharmthera.2015.01.009 25601315 PMC4414735

[82] Ramboz S Oosting R Amara DA Kung HF Blier P Mendelsohn M Serotonin receptor 1A knockout: an animal model of anxiety-related disorder. Proc Natl Acad Sci U S A (1998) 95(24):14476–81. 10.1073/pnas.95.24.14476 9826725 PMC24398

[83] Toth M. 5-HT1A receptor knockout mouse as a genetic model of anxiety. Eur J Pharmacol (2003) 463(1–3):177–84. 10.1016/S0014-2999(03)01280-9 12600709

[84] Popova NK Naumenko VS. 5-HT1A receptor as a key player in the brain 5-HT system. Rev Neurosci (2013) 24(2):191–204. 10.1515/revneuro-2012-0082 23492554

[85] Launay JM Schneider B Loric S Da Prada M Kellermann O. Serotonin transport and serotonin transporter-mediated antidepressant recognition are controlled by 5-HT2B receptor signaling in serotonergic neuronal cells. FASEB J (2006) 20(11):1843–54. 10.1096/fj.06-5724com 16940156

[86] Doly S Valjent E Setola V Callebert J Herve D Launay JM Serotonin 5-HT2B receptors are required for 3,4-methylenedioxymethamphetamine-induced hyperlocomotion and 5-HT release in vivo and in vitro. J Neurosci (2008) 28(11):2933–40. 10.1523/JNEUROSCI.5723-07.2008 18337424 PMC6670669

[87] Singer HS Minzer K. Neurobiology of Tourette’s syndrome: concepts of neuroanatomic localization and neurochemical abnormalities. Brain Dev (2003) 25(Suppl 1):S70–84. 10.1016/S0387-7604(03)90012-X 14980376

[88] Adamczyk A Gause CD Sattler R Vidensky S Rothstein JD Singer H Genetic and functional studies of a missense variant in a glutamate transporter, SLC1A3, in Tourette syndrome. Psychiatr Genet (2011) 21(2):90–7. 10.1097/YPG.0b013e328341a307 21233784

[89] Manev H Favaron M Guidotti A Costa E. Delayed increase of Ca2+ influx elicited by glutamate: role in neuronal death. Mol Pharmacol (1989) 36(1):106–12. 2568579

[90] O’Shea RD. Roles and regulation of glutamate transporters in the central nervous system. Clin Exp Pharmacol Physiol (2002) 29(11):1018–23. 10.1046/j.1440-1681.2002.03770.x 12366395

[91] Furuta A Rothstein JD Martin LJ. Glutamate transporter protein subtypes are expressed differentially during rat CNS development. J Neurosci (1997) 17(21):8363–75. 9334410 10.1523/JNEUROSCI.17-21-08363.1997 PMC6573756

[92] Karlsson RM Tanaka K Saksida LM Bussey TJ Heilig M Holmes A. Assessment of glutamate transporter GLAST (EAAT1)-deficient mice for phenotypes relevant to the negative and executive/cognitive symptoms of schizophrenia. Neuropsychopharmacology (2009) 34(6):1578–89. 10.1038/npp.2008.215 19078949 PMC3400972

[93] Zebardast N Crowley MJ Bloch MH Mayes LC Wyk BV Leckman JF Brain mechanisms for prepulse inhibition in adults with Tourette syndrome: initial findings. Psychiatry Res (2013) 214(1):33–41. 10.1016/j.pscychresns.2013.05.009 23916249 PMC3932431

[94] Zhang C Milunsky JM Newton S Ko J Zhao G Maher TA A neuroligin-4 missense mutation associated with autism impairs neuroligin-4 folding and endoplasmic reticulum export. J Neurosci (2009) 29(35):10843–54. 10.1523/JNEUROSCI.1248-09.2009 19726642 PMC2777970

[95] Marshall CR Noor A Vincent JB Lionel AC Feuk L Skaug J Structural variation of chromosomes in autism spectrum disorder. Am J Hum Genet (2008) 82(2):477–88. 10.1016/j.ajhg.2007.12.009 18252227 PMC2426913

[96] Laumonnier F Bonnet-Brilhault F Gomot M Blanc R David A Moizard MP X-linked mental retardation and autism are associated with a mutation in the NLGN4 gene, a member of the neuroligin family. Am J Hum Genet (2004) 74(3):552–7. 10.1086/382137 14963808 PMC1182268

[97] Jamain S Quach H Betancur C Rastam M Colineaux C Gillberg IC Mutations of the X-linked genes encoding neuroligins NLGN3 and NLGN4 are associated with autism. Nat Genet (2003) 34(1):27–9. 10.1038/ng1136 12669065 PMC1925054

[98] Kim JE O’Sullivan ML Sanchez CA Hwang M Israel MA Brennand K Investigating synapse formation and function using human pluripotent stem cell-derived neurons. Proc Natl Acad Sci U S A (2011) 108(7):3005–10. 10.1073/pnas.1007753108 21278334 PMC3041068

[99] Lawson-Yuen A Saldivar JS Sommer S Picker J. Familial deletion within NLGN4 associated with autism and Tourette syndrome. Eur J Hum Genet (2008) 16(5):614–8. 10.1038/sj.ejhg.5202006 18231125

[100] Sudhof TC. Neuroligins and neurexins link synaptic function to cognitive disease. Nature (2008) 455(7215):903–11. 10.1038/nature07456 18923512 PMC2673233

[101] Varoqueaux F Aramuni G Rawson RL Mohrmann R Missler M Gottmann K Neuroligins determine synapse maturation and function. Neuron (2006) 51(6):741–54. 10.1016/j.neuron.2006.09.003 16982420

[102] Chih B Engelman H Scheiffele P. Control of excitatory and inhibitory synapse formation by neuroligins. Science (2005) 307(5713):1324–8. 10.1126/science.1107470 15681343

[103] Jamain S Radyushkin K Hammerschmidt K Granon S Boretius S Varoqueaux F Reduced social interaction and ultrasonic communication in a mouse model of monogenic heritable autism. Proc Natl Acad Sci U S A (2008) 105(5):1710–5. 10.1073/pnas.0711555105 18227507 PMC2234209

[104] Delattre V La Mendola D Meystre J Markram H Markram K. Nlgn4 knockout induces network hypo-excitability in juvenile mouse somatosensory cortex in vitro. Sci Rep (2013) 3:2897. 10.1038/srep02897 24104404 PMC3793213

[105] El-Kordi A Winkler D Hammerschmidt K Kastner A Krueger D Ronnenberg A Development of an autism severity score for mice using Nlgn4 null mutants as a construct-valid model of heritable monogenic autism. Behav Brain Res (2013) 251:41–9. 10.1016/j.bbr.2012.11.016 23183221

[106] Moya PR Dodman NH Timpano KR Rubenstein LM Rana Z Fried RL Rare missense neuronal cadherin gene (CDH2) variants in specific obsessive-compulsive disorder and Tourette disorder phenotypes. Eur J Hum Genet (2013) 21(8):850–4. 10.1038/ejhg.2012.245 23321619 PMC3722668

[107] Hatta K Okada TS Takeichi M. A monoclonal antibody disrupting calcium-dependent cell-cell adhesion of brain tissues: possible role of its target antigen in animal pattern formation. Proc Natl Acad Sci U S A (1985) 82(9):2789–93. 10.1073/pnas.82.9.2789 3857614 PMC397651

[108] Reichardt LFN- Cadherin and integrins: two receptor systems that mediate neuronal process outgrowth on astrocyte surfaces. Neuron (2008) 60(3):398–9. 10.1016/j.neuron.2008.10.030 18995807 PMC2623248

[109] Tomaselli KJ Neugebauer KM Bixby JL Lilien J Reichardt LF. N-cadherin and integrins: two receptor systems that mediate neuronal process outgrowth on astrocyte surfaces. Neuron (1988) 1(1):33–43. 10.1016/0896-6273(88)90207-3 2856086

[110] Arikkath J Reichardt LF. Cadherins and catenins at synapses: roles in synaptogenesis and synaptic plasticity. Trends Neurosci (2008) 31(9):487–94. 10.1016/j.tins.2008.07.001 18684518 PMC2623250

[111] Bamji SX Shimazu K Kimes N Huelsken J Birchmeier W Lu B Role of beta-catenin in synaptic vesicle localization and presynaptic assembly. Neuron (2003) 40(4):719–31. 10.1016/S0896-6273(03)00718-9 14622577 PMC2757419

[112] Okuda T Yu LM Cingolani LA Kemler R Goda Y. beta-Catenin regulates excitatory postsynaptic strength at hippocampal synapses. Proc Natl Acad Sci U S A (2007) 104(33):13479–84. 10.1073/pnas.0702334104 17679699 PMC1948936

[113] Marambaud P Wen PH Dutt A Shioi J Takashima A Siman R A CBP binding transcriptional repressor produced by the PS1/epsilon-cleavage of N-cadherin is inhibited by PS1 FAD mutations. Cell (2003) 114(5):635–45. 10.1016/j.cell.2003.08.008 13678586

[114] Radice GL Rayburn H Matsunami H Knudsen KA Takeichi M Hynes RO. Developmental defects in mouse embryos lacking N-cadherin. Dev Biol (1997) 181(1):64–78. 10.1006/dbio.1996.8443 9015265

[115] Kadowaki M Nakamura S Machon O Krauss S Radice GL Takeichi M N-cadherin mediates cortical organization in the mouse brain. Dev Biol (2007) 304(1):22–33. 10.1016/j.ydbio.2006.12.014 17222817

[116] Arking DE Cutler DJ Brune CW Teslovich TM West K Ikeda M A common genetic variant in the neurexin superfamily member CNTNAP2 increases familial risk of autism. Am J Hum Genet (2008) 82(1):160–4. 10.1016/j.ajhg.2007.09.015 18179894 PMC2253968

[117] Alarcon M Abrahams BS Stone JL Duvall JA Perederiy JV Bomar JM Linkage, association, and gene-expression analyses identify CNTNAP2 as an autism-susceptibility gene. Am J Hum Genet (2008) 82(1):150–9. 10.1016/j.ajhg.2007.09.005 18179893 PMC2253955

[118] Strauss KA Puffenberger EG Huentelman MJ Gottlieb S Dobrin SE Parod JM Recessive symptomatic focal epilepsy and mutant contactin-associated protein-like 2. N Engl J Med (2006) 354(13):1370–7. 10.1056/NEJMoa052773 16571880

[119] Penagarikano O Geschwind DH. What does CNTNAP2 reveal about autism spectrum disorder? Trends Mol Med (2012) 18(3):156–63. 10.1016/j.molmed.2012.01.003 22365836 PMC3633421

[120] Bakkaloglu B O’Roak BJ Louvi A Gupta AR Abelson JF Morgan TM Molecular cytogenetic analysis and resequencing of contactin associated protein-like 2 in autism spectrum disorders. Am J Hum Genet (2008) 82(1):165–73. 10.1016/j.ajhg.2007.09.017 18179895 PMC2253974

[121] Verkerk AJ Mathews CA Joosse M Eussen BH Heutink P Oostra BA. CNTNAP2 is disrupted in a family with Gilles de la Tourette syndrome and obsessive compulsive disorder. Genomics (2003) 82(1):1–9. 10.1016/S0888-7543(03)00097-1 12809671

[122] Belloso JM Bache I Guitart M Caballin MR Halgren C Kirchhoff M Disruption of the CNTNAP2 gene in a t(7;15) translocation family without symptoms of Gilles de la Tourette syndrome. Eur J Hum Genet (2007) 15(6):711–3. 10.1038/sj.ejhg.5201824 17392702

[123] Poliak S Gollan L Martinez R Custer A Einheber S Salzer JL Caspr2, a new member of the neurexin superfamily, is localized at the juxtaparanodes of myelinated axons and associates with K+ channels. Neuron (1999) 24(4):1037–47. 10.1016/S0896-6273(00)81049-1 10624965

[124] Bhat MA Rios JC Lu Y Garcia-Fresco GP Ching W St Martin M Axon-glia interactions and the domain organization of myelinated axons requires neurexin IV/Caspr/Paranodin. Neuron (2001) 30(2):369–83. 10.1016/S0896-6273(01)00294-X 11395000

[125] Mink JW. Basal ganglia dysfunction in Tourette’s syndrome: a new hypothesis. Pediatr Neurol (2001) 25(3):190–8. 10.1016/S0887-8994(01)00262-4 11587872

[126] Mink JW Neurobiology of basal ganglia circuits in Tourette syndrome: faulty inhibition of unwanted motor patterns? Adv Neurol (2001) 85:113–22. 11530421

[127] Penagarikano O Abrahams BS Herman EI Winden KD Gdalyahu A Dong H Absence of CNTNAP2 leads to epilepsy, neuronal migration abnormalities, and core autism-related deficits. Cell (2011) 147(1):235–46. 10.1016/j.cell.2011.08.040 21962519 PMC3390029

[128] Prontera P Napolioni V Ottaviani V Rogaia D Fusco C Augello B DPP6 gene disruption in a family with Gilles de la Tourette syndrome. Neurogenetics (2014) 15(4):237–42. 10.1007/s10048-014-0418-9 25129042

[129] Egger G Roetzer KM Noor A Lionel AC Mahmood H Schwarzbraun T Identification of risk genes for autism spectrum disorder through copy number variation analysis in Austrian families. Neurogenetics (2014) 15(2):117–27. 10.1007/s10048-014-0394-0 24643514

[130] Tanaka S Syu A Ishiguro H Inada T Horiuchi Y Ishikawa M DPP6 as a candidate gene for neuroleptic-induced tardive dyskinesia. Pharmacogenomics J (2013) 13(1):27–34. 10.1038/tpj.2011.36 21826085

[131] Kin Y Misumi Y Ikehara Y. Biosynthesis and characterization of the brain-specific membrane protein DPPX, a dipeptidyl peptidase IV-related protein. J Biochem (2001) 129(2):289–95. 10.1093/oxfordjournals.jbchem.a002856 11173531

[132] Nadal MS Amarillo Y Vega-Saenz de Miera E Rudy B Differential characterization of three alternative spliced isoforms of DPPX. Brain Res (2006) 1094(1):1–12. 10.1016/j.brainres.2006.03.106 16764835

[133] Strop P Bankovich AJ Hansen KC Garcia KC Brunger AT. Structure of a human A-type potassium channel interacting protein DPPX, a member of the dipeptidyl aminopeptidase family. J Mol Biol (2004) 343(4):1055–65. 10.1016/j.jmb.2004.09.003 15476821

[134] Clark BD Kwon E Maffie J Jeong HY Nadal M Strop P DPP6 localization in brain supports function as a Kv4 channel associated protein. Front Mol Neurosci (2008) 1:8. 10.3389/neuro.02.008.2008 18978958 PMC2576564

[135] Schoppa NE Westbrook GL. Regulation of synaptic timing in the olfactory bulb by an A-type potassium current. Nat Neurosci (1999) 2(12):1106–13. 10.1038/16033 10570488

[136] Johnston D Hoffman DA Magee JC Poolos NP Watanabe S Colbert CM Dendritic potassium channels in hippocampal pyramidal neurons. J Physiol (2000) 525(Pt 1):75–81. 10.1111/j.1469-7793.2000.00075.x 10811726 PMC2269937

[137] Liss B Franz O Sewing S Bruns R Neuhoff H Roeper J. Tuning pacemaker frequency of individual dopaminergic neurons by Kv4.3L and KChip3.1 transcription. EMBO J (2001) 20(20):5715–24. 10.1093/emboj/20.20.5715 11598014 PMC125678

[138] Nadal MS Ozaita A Amarillo Y Vega-Saenz de Miera E Ma Y Mo W The CD26-related dipeptidyl aminopeptidase-like protein DPPX is a critical component of neuronal A-type K+ channels. Neuron (2003) 37(3):449–61. 10.1016/S0896-6273(02)01185-6 12575952

[139] Lin L Sun W Throesch B Kung F Decoster JT Berner CJ DPP6 regulation of dendritic morphogenesis impacts hippocampal synaptic development. Nat Commun (2013) 4:2270. 10.1038/ncomms3270 23912628 PMC3775611

[140] Liao C Fu F Li R Yang WQ Liao HY Yan JR Loss-of-function variation in the DPP6 gene is associated with autosomal dominant microcephaly and mental retardation. Eur J Med Genet (2013) 56(9):484–9. 10.1016/j.ejmg.2013.06.008 23832105

[141] Boghosian-Sell L Comings DE Overhauser J. Tourette syndrome in a pedigree with a 7;18 translocation: identification of a YAC spanning the translocation breakpoint at 18q22.3. Am J Hum Genet (1996) 59(5):999–1005. 8900226 PMC1914824

[142] Petek E Windpassinger C Vincent JB Cheung J Boright AP Scherer SW Disruption of a novel gene (IMMP2L) by a breakpoint in 7q31 associated with Tourette syndrome. Am J Hum Genet (2001) 68(4):848–58. 10.1086/319523 11254443 PMC1275638

[143] Patel C Cooper-Charles L McMullan DJ Walker JM Davison V Morton J. Translocation breakpoint at 7q31 associated with tics: further evidence for IMMP2L as a candidate gene for Tourette syndrome. Eur J Hum Genet (2011) 19(6):634–9. 10.1038/ejhg.2010.238 21386874 PMC3110039

[144] Bertelsen B Melchior L Jensen LR Groth C Glenthoj B Rizzo R Intragenic deletions affecting two alternative transcripts of the IMMP2L gene in patients with Tourette syndrome. Eur J Hum Genet (2014) 22(11):1283–9. 10.1038/ejhg.2014.24 24549057 PMC4200436

[145] Gakh O Cavadini P Isaya G. Mitochondrial processing peptidases. Biochim Biophys Acta (2002) 1592(1):63–77. 10.1016/S0167-4889(02)00265-3 12191769

[146] Burri L Strahm Y Hawkins CJ Gentle IE Puryer MA Verhagen A Mature DIABLO/Smac is produced by the IMP protease complex on the mitochondrial inner membrane. Mol Biol Cell (2005) 16(6):2926–33. 10.1091/mbc.E04-12-1086 15814844 PMC1142436

[147] Nunnari J Fox TD Walter P. A mitochondrial protease with two catalytic subunits of nonoverlapping specificities. Science (1993) 262(5142):1997–2004. 10.1126/science.8266095 8266095

[148] Lu B Poirier C Gaspar T Gratzke C Harrison W Busija D A mutation in the inner mitochondrial membrane peptidase 2-like gene (Immp2l) affects mitochondrial function and impairs fertility in mice. Biol Reprod (2008) 78(4):601–10. 10.1095/biolreprod.107.065987 18094351

[149] Ma Y Mehta SL Lu B Li PA. Deficiency in the inner mitochondrial membrane peptidase 2-like (Immp21) gene increases ischemic brain damage and impairs mitochondrial function. Neurobiol Dis (2011) 44(3):270–6. 10.1016/j.nbd.2011.06.019 21824519 PMC3185146

[150] Sundaram SK Huq AM Sun Z Yu W Bennett L Wilson BJ Exome sequencing of a pedigree with Tourette syndrome or chronic tic disorder. Ann Neurol (2011) 69(5):901–4. 10.1002/ana.22398 21520241

[151] Guo Y Deng X Zhang J Su L Xu H Luo Z Analysis of the MRPL3, DNAJC13 and OFCC1 variants in Chinese Han patients with TS-CTD. Neurosci Lett (2012) 517(1):18–20. 10.1016/j.neulet.2012.03.097 22507240

[152] Gruschke S Grone K Heublein M Holz S Israel L Imhof A Proteins at the polypeptide tunnel exit of the yeast mitochondrial ribosome. J Biol Chem (2010) 285(25):19022–8. 10.1074/jbc.M110.113837 20404317 PMC2885179

[153] Galmiche L Serre V Beinat M Assouline Z Lebre AS Chretien D Exome sequencing identifies MRPL3 mutation in mitochondrial cardiomyopathy. Hum Mutat (2011) 32(11):1225–31. 10.1002/humu.21562 21786366

[154] Vilarino-Guell C Rajput A Milnerwood AJ Shah B Szu-Tu C Trinh J DNAJC13 mutations in Parkinson disease. Hum Mol Genet (2014) 23(7):1794–801. 10.1093/hmg/ddt570 24218364 PMC3999380

[155] Girard M Poupon V Blondeau F McPherson PS. The DnaJ-domain protein RME-8 functions in endosomal trafficking. J Biol Chem (2005) 280(48):40135–43. 10.1074/jbc.M505036200 16179350

[156] Fujibayashi A Taguchi T Misaki R Ohtani M Dohmae N Takio K Human RME-8 is involved in membrane trafficking through early endosomes. Cell Struct Funct (2008) 33(1):35–50. 10.1247/csf.07045 18256511

[157] Davies SJ Wise C Venkatesh B Mirza G Jefferson A Volpi EV Mapping of three translocation breakpoints associated with orofacial clefting within 6p24 and identification of new transcripts within the region. Cytogenet Genome Res (2004) 105(1):47–53. 10.1159/000078008 15218257

[158] Ohnishi T Yamada K Watanabe A Ohba H Sakaguchi T Honma Y Ablation of Mrds1/Ofcc1 induces hyper-gamma-glutamyl transpeptidasemia without abnormal head development and schizophrenia-relevant behaviors in mice. PLoS One (2011) 6(12):e29499 10.1371/journal.pone.0029499 22242126 PMC3248446

[159] Mattheisen M Samuels JF Wang Y Greenberg BD Fyer AJ McCracken JT Genome-wide association study in obsessive-compulsive disorder: results from the OCGAS. Mol Psychiatry (2015) 20:337–44. 10.1038/mp.2014.43 24821223 PMC4231023

[160] Cukier HN Dueker ND Slifer SH Lee JM Whitehead PL Lalanne E Exome sequencing of extended families with autism reveals genes shared across neurodevelopmental and neuropsychiatric disorders. Mol Autism (2014) 5(1):1. 10.1186/2040-2392-5-1 24410847 PMC3896704

[161] Weimann M Grossmann A Woodsmith J Ozkan Z Birth P Meierhofer D A Y2H-seq approach defines the human protein methyltransferase interactome. Nat Methods (2013) 10(4):339–42. 10.1038/nmeth.2397 23455924

[162] Thompson MD Comings DE Abu-Ghazalah R Jereseh Y Lin L Wade J Variants of the orexin2/hcrt2 receptor gene identified in patients with excessive daytime sleepiness and patients with Tourette’s syndrome comorbidity. Am J Med Genet B Neuropsychiatr Genet (2004) 129B(1):69–75. 10.1002/ajmg.b.30047 15274044

[163] Willie JT Chemelli RM Sinton CM Yanagisawa M. To eat or to sleep? Orexin in the regulation of feeding and wakefulness. Annu Rev Neurosci (2001) 24:429–58. 10.1146/annurev.neuro.24.1.429 11283317

[164] Tsujino N Sakurai T. Role of orexin in modulating arousal, feeding, and motivation. Front Behav Neurosci (2013) 7:28. 10.3389/fnbeh.2013.00028 23616752 PMC3629303

[165] Geschwind DH. Autism: many genes, common pathways? Cell (2008) 135(3):391–5. 10.1016/j.cell.2008.10.016 18984147 PMC2756410

[166] Gossen M Bujard H. Tight control of gene expression in mammalian cells by tetracycline-responsive promoters. Proc Natl Acad Sci U S A (1992) 89(12):5547–51. 10.1073/pnas.89.12.5547 1319065 PMC49329

[167] Mele M Ferreira PG Reverter F DeLuca DS Monlong J Sammeth M Human genomics. The human transcriptome across tissues and individuals. Science (2015) 348(6235):660–5. 10.1126/science.aaa0355 25954002 PMC4547472

[168] Takahashi K Yamanaka S. Induction of pluripotent stem cells from mouse embryonic and adult fibroblast cultures by defined factors. Cell (2006) 126(4):663–76. 10.1016/j.cell.2006.07.024 16904174

[169] Young-Pearse TL Morrow EM. Modeling developmental neuropsychiatric disorders with iPSC technology: challenges and opportunities. Curr Opin Neurobiol (2015) 36:66–73. 10.1016/j.conb.2015.10.006 26517284 PMC4738093

[170] Amir RE Van den Veyver IB Wan M Tran CQ Francke U Zoghbi HY. Rett syndrome is caused by mutations in X-linked MECP2, encoding methyl-CpG-binding protein 2. Nat Genet (1999) 23(2):185–8. 10.1038/13810 10508514

[171] Cheung AY Horvath LM Grafodatskaya D Pasceri P Weksberg R Hotta A Isolation of MECP2-null Rett syndrome patient hiPS cells and isogenic controls through X-chromosome inactivation. Hum Mol Genet (2011) 20(11):2103–15. 10.1093/hmg/ddr093 21372149 PMC3090191

[172] Griesi-Oliveira K Acab A Gupta AR Sunaga DY Chailangkarn T Nicol X Modeling non-syndromic autism and the impact of TRPC6 disruption in human neurons. Mol Psychiatry (2015) 20(11):1350–65. 10.1038/mp.2014.141 25385366 PMC4427554

[173] Wen Z Nguyen HN Guo Z Lalli MA Wang X Su Y Synaptic dysregulation in a human iPS cell model of mental disorders. Nature (2014) 515(7527):414–8. 10.1038/nature13716 25132547 PMC4501856

[174] Egawa N Kitaoka S Tsukita K Naitoh M Takahashi K Yamamoto T Drug screening for ALS using patient-specific induced pluripotent stem cells. Sci Transl Med (2012) 4(145):145ra04. 10.1126/scitranslmed.3004052 22855461

[175] Thomson PA Malavasi EL Grunewald E Soares DC Borkowska M Millar JK. DISC1 genetics, biology and psychiatric illness. Front Biol (2013) 8(1):1–31. 10.1007/s11515-012-1254-7 23550053 PMC3580875

[176] Hornykiewicz O Brain monoamines and Parkinsonism. Psychopharmacol Bull (1975) 11(3):34–5. 1153656

[177] Jiang H Ren Y Yuen EY Zhong P Ghaedi M Hu Z Parkin controls dopamine utilization in human midbrain dopaminergic neurons derived from induced pluripotent stem cells. Nat Commun (2012) 3:668. 10.1038/ncomms1669 22314364 PMC3498452

[178] Beevers JE Caffrey TM Wade-Martins R. Induced pluripotent stem cell (iPSC)-derived dopaminergic models of Parkinson’s disease. Biochem Soc Trans (2013) 41(6):1503–8. 10.1042/BST20130194 24256244

[179] Su YC Qi X. Inhibition of excessive mitochondrial fission reduced aberrant autophagy and neuronal damage caused by LRRK2 G2019S mutation. Hum Mol Genet (2013) 22(22):4545–61. 10.1093/hmg/ddt301 23813973

[180] Reinhardt P Schmid B Burbulla LF Schondorf DC Wagner L Glatza M Genetic correction of a LRRK2 mutation in human iPSCs links parkinsonian neurodegeneration to ERK-dependent changes in gene expression. Cell Stem Cell (2013) 12(3):354–67. 10.1016/j.stem.2013.01.008 23472874

[181] Chen H Qian K Du Z Cao J Petersen A Liu H Modeling ALS with iPSCs reveals that mutant SOD1 misregulates neurofilament balance in motor neurons. Cell Stem Cell (2014) 14(6):796–809. 10.1016/j.stem.2014.02.004 24704493 PMC4230530

[182] Nguyen HN Byers B Cord B Shcheglovitov A Byrne J Gujar P LRRK2 mutant iPSC-derived DA neurons demonstrate increased susceptibility to oxidative stress. Cell Stem Cell (2011) 8(3):267–80. 10.1016/j.stem.2011.01.013 21362567 PMC3578553

[183] Pasca SP Portmann T Voineagu I Yazawa M Shcheglovitov A Pasca AM Using iPSC-derived neurons to uncover cellular phenotypes associated with Timothy syndrome. Nat Med (2011) 17(12):1657–62. 10.1038/nm.2576 22120178 PMC3517299

[184] White AK VanInsberghe M Petriv OI Hamidi M Sikorski D Marra MA High-throughput microfluidic single-cell RT-qPCR. Proc Natl Acad Sci U S A (2011) 108(34):13999–4004. 10.1073/pnas.1019446108 21808033 PMC3161570

[185] White AK Heyries KA Doolin C Vaninsberghe M Hansen CL. High-throughput microfluidic single-cell digital polymerase chain reaction. Anal Chem (2013) 85(15):7182–90. 10.1021/ac400896j 23819473

[186] Lancaster MA Knoblich JA. Generation of cerebral organoids from human pluripotent stem cells. Nat Protoc (2014) 9(10):2329–40. 10.1038/nprot.2014.158 25188634 PMC4160653

[187] Lancaster MA Renner M Martin CA Wenzel D Bicknell LS Hurles ME Cerebral organoids model human brain development and microcephaly. Nature (2013) 501(7467):373–9. 10.1038/nature12517 23995685 PMC3817409

[188] Mariani J Coppola G Zhang P Abyzov A Provini L Tomasini L FOXG1-dependent dysregulation of GABA/glutamate neuron differentiation in autism spectrum disorders. Cell (2015) 162(2):375–90. 10.1016/j.cell.2015.06.034 26186191 PMC4519016

[189] Bobis-Wozowicz S Osiak A Rahman SH Cathomen T. Targeted genome editing in pluripotent stem cells using zinc-finger nucleases. Methods (2011) 53(4):339–46. 10.1016/j.ymeth.2010.12.019 21185378

[190] Soldner F Laganiere J Cheng AW Hockemeyer D Gao Q Alagappan R Generation of isogenic pluripotent stem cells differing exclusively at two early onset Parkinson point mutations. Cell (2011) 146(2):318–31. 10.1016/j.cell.2011.06.019 21757228 PMC3155290

[191] Smith C Abalde-Atristain L He C Brodsky BR Braunstein EM Chaudhari P Efficient and allele-specific genome editing of disease loci in human iPSCs. Mol Ther (2015) 23(3):570–7. 10.1038/mt.2014.226 25418680 PMC4351458

[192] Byrne SM Mali P Church GM. Genome editing in human stem cells. Methods Enzymol (2014) 546:119–38. 10.1016/B978-0-12-801185-0.00006-4 25398338 PMC4408990

[193] Fu Y Foden JA Khayter C Maeder ML Reyon D Joung JK High-frequency off-target mutagenesis induced by CRISPR-Cas nucleases in human cells. Nat Biotechnol (2013) 31(9):822–6. 10.1038/nbt.2623 23792628 PMC3773023

[194] Costa V Aprile M Esposito R Ciccodicola A. RNA-Seq and human complex diseases: recent accomplishments and future perspectives. Eur J Hum Genet (2013) 21(2):134–42. 10.1038/ejhg.2012.129 22739340 PMC3548270

[195] Marchetto MC Brennand KJ Boyer LF Gage FH. Induced pluripotent stem cells (iPSCs) and neurological disease modeling: progress and promises. Hum Mol Genet (2011) 20(R2):R109–15. 10.1093/hmg/ddr336 21828073 PMC4447776

[196] Soneson C Delorenzi M. A comparison of methods for differential expression analysis of RNA-seq data. BMC Bioinformatics (2013) 14:91. 10.1186/1471-2105-14-91 23497356 PMC3608160

[197] Trapnell C Hendrickson DG Sauvageau M Goff L Rinn JL Pachter L. Differential analysis of gene regulation at transcript resolution with RNA-seq. Nat Biotechnol (2013) 31(1):46–53. 10.1038/nbt.2450 23222703 PMC3869392

[198] Trapnell C Williams BA Pertea G Mortazavi A Kwan G van Baren MJ Transcript assembly and quantification by RNA-Seq reveals unannotated transcripts and isoform switching during cell differentiation. Nat Biotechnol (2010) 28(5):511–5. 10.1038/nbt.1621 20436464 PMC3146043

[199] Kanehisa M Goto S Sato Y Kawashima M Furumichi M Tanabe M. Data, information, knowledge and principle: back to metabolism in KEGG. Nucleic Acids Res (2014) 42(Database issue):D199–205. 10.1093/nar/gkt1076 24214961 PMC3965122

[200] Kanehisa M Goto S. KEGG: kyoto encyclopedia of genes and genomes. Nucleic Acids Res (2000) 28(1):27–30. 10.1093/nar/28.1.27 10592173 PMC102409

[201] Huang da W Sherman BT Lempicki RA. Systematic and integrative analysis of large gene lists using DAVID bioinformatics resources. Nat Protoc (2009) 4(1):44–57. 10.1038/nprot.2008.211 19131956

[202] Huang da W Sherman BT Lempicki RA. Bioinformatics enrichment tools: paths toward the comprehensive functional analysis of large gene lists. Nucleic Acids Res (2009) 37(1):1–13. 10.1093/nar/gkn923 19033363 PMC2615629

[203] Kamburov A Wierling C Lehrach H Herwig R ConsensusPathDB – a database for integrating human functional interaction networks. Nucleic Acids Res (2009) 37(Database issue):D623–8. 10.1093/nar/gkn698 18940869 PMC2686562

[204] Parikshak NN Gandal MJ Geschwind DH. Systems biology and gene networks in neurodevelopmental and neurodegenerative disorders. Nat Rev Genet (2015) 16(8):441–58. 10.1038/nrg3934 26149713 PMC4699316

[205] Hawrylycz MJ Lein ES Guillozet-Bongaarts AL Shen EH Ng L Miller JA An anatomically comprehensive atlas of the adult human brain transcriptome. Nature (2012) 489(7416):391–9. 10.1038/nature11405 22996553 PMC4243026

[206] Kang HJ Kawasawa YI Cheng F Zhu Y Xu X Li M Spatio-temporal transcriptome of the human brain. Nature (2011) 478(7370):483–9. 10.1038/nature10523 22031440 PMC3566780

[207] Keen JC Moore HM. The genotype-tissue expression (GTEx) project: linking clinical data with molecular analysis to advance personalized medicine. J Pers Med (2015) 5(1):22–9. 10.3390/jpm5010022 25809799 PMC4384056

[208] Cotney J Muhle RA Sanders SJ Liu L Willsey AJ Niu W The autism-associated chromatin modifier CHD8 regulates other autism risk genes during human neurodevelopment. Nat Commun (2015) 6:6404. 10.1038/ncomms7404 25752243 PMC4355952

[209] Darmanis S Sloan SA Zhang Y Enge M Caneda C Shuer LM A survey of human brain transcriptome diversity at the single cell level. Proc Natl Acad Sci U S A (2015) 112(23):7285–90. 10.1073/pnas.1507125112 26060301 PMC4466750

[210] Stark C Breitkreutz BJ Reguly T Boucher L Breitkreutz A Tyers M. BioGRID: a general repository for interaction datasets. Nucleic Acids Res (2006) 34(Database issue):D535–9. 10.1093/nar/gkj109 16381927 PMC1347471

[211] Gilman SR Iossifov I Levy D Ronemus M Wigler M Vitkup D. Rare de novo variants associated with autism implicate a large functional network of genes involved in formation and function of synapses. Neuron (2011) 70(5):898–907. 10.1016/j.neuron.2011.05.021 21658583 PMC3607702

[212] Willsey AJ Sanders SJ Li M Dong S Tebbenkamp AT Muhle RA Coexpression networks implicate human midfetal deep cortical projection neurons in the pathogenesis of autism. Cell (2013) 155(5):997–1007. 10.1016/j.cell.2013.10.020 24267886 PMC3995413

[213] Chang J Gilman SR Chiang AH Sanders SJ Vitkup D. Genotype to phenotype relationships in autism spectrum disorders. Nat Neurosci (2015) 18(2):191–8. 10.1038/nn.3907 25531569 PMC4397214

[214] Platt RJ Chen S Zhou Y Yim MJ Swiech L Kempton HR CRISPR-Cas9 knockin mice for genome editing and cancer modeling. Cell (2014) 159(2):440–55. 10.1016/j.cell.2014.09.014 25263330 PMC4265475

[215] Yang H Wang H Shivalila CS Cheng AW Shi L Jaenisch R. One-step generation of mice carrying reporter and conditional alleles by CRISPR/Cas-mediated genome engineering. Cell (2013) 154(6):1370–9. 10.1016/j.cell.2013.08.022 23992847 PMC3961003

[216] Godar SC Mosher LJ Di Giovanni G Bortolato M. Animal models of tic disorders: a translational perspective. J Neurosci Methods (2014) 238:54–69. 10.1016/j.jneumeth.2014.09.008 25244952 PMC4345166

[217] Comings DE Wu S Chiu C Ring RH Gade R Ahn C Polygenic inheritance of Tourette syndrome, stuttering, attention deficit hyperactivity, conduct, and oppositional defiant disorder: the additive and subtractive effect of the three dopaminergic genes – DRD2, D beta H, and DAT1. Am J Med Genet (1996) 67(3):264–88. 10.1002/(SICI)1096-8628(19960531)67:3<264::AID-AJMG4>3.0.CO;2-N 8725745

[218] Paschou P. The genetic basis of Gilles de la Tourette syndrome. Neurosci Biobehav Rev (2013) 37(6):1026–39. 10.1016/j.neubiorev.2013.01.016 23333760

[219] Clarke RA Lee S Eapen V. Pathogenetic model for Tourette syndrome delineates overlap with related neurodevelopmental disorders including autism. Transl Psychiatry (2012) 2:e158. 10.1038/tp.2012.75 22948383 PMC3565204

[220] Yee CM Javitt DC Miller GA Replacing DSM categorical analyses with dimensional analyses in psychiatry research: the research domain criteria initiative. JAMA Psychiatry (2015) 72(12):1159–60. 10.1001/jamapsychiatry.2015.1900 26559005

[221] Kraemer HC Research domain criteria (RDoC) and the DSM-two methodological approaches to mental health diagnosis. JAMA Psychiatry (2015) 72(12):1163–4. 10.1001/jamapsychiatry.2015.2134 26559143

[222] Insel T Cuthbert B Garvey M Heinssen R Pine DS Quinn K Research domain criteria (RDoC): toward a new classification framework for research on mental disorders. Am J Psychiatry (2010) 167(7):748–51. 10.1176/appi.ajp.2010.09091379 20595427

[223] Casey BJ Oliveri ME Insel T A neurodevelopmental perspective on the research domain criteria (RDoC) framework. Biol Psychiatry (2014) 76(5):350–3. 10.1016/j.biopsych.2014.01.006 25103538

[224] Cuthbert BN. Research domain criteria: toward future psychiatric nosologies. Dialogues Clin Neurosci (2015) 17(1):89–97. 25987867 10.31887/DCNS.2015.17.1/bcuthbert PMC4421905

[225] Heiman GA King RA Tischfield JA. New Jersey center for Tourette syndrome sharing repository: methods and sample description. BMC Med Genomics (2008) 1:58. 10.1186/1755-8794-1-58 19036136 PMC2605751

[226] Dietrich A Fernandez TV King RA State MW Tischfield JA Hoekstra PJ The Tourette International Collaborative Genetics (TIC Genetics) study, finding the genes causing Tourette syndrome: objectives and methods. Eur Child Adolesc Psychiatry (2015) 24(2):141–51. 10.1007/s00787-014-0543-x 24771252 PMC4209328

